# CGAT: a comparative genome analysis tool for visualizing alignments in the analysis of complex evolutionary changes between closely related genomes

**DOI:** 10.1186/1471-2105-7-472

**Published:** 2006-10-24

**Authors:** Ikuo Uchiyama, Toshio Higuchi, Ichizo Kobayashi

**Affiliations:** 1 National Institute for Basic Biology, National Institutes of Natural Sciences, Nishigonaka 38, Myodaiji, Okazaki, Aichi 444-8585, Japan; 2 INTEC Web and Genome Informatics Corporation, 1-3-3 Shinsuna, Koto-ku, Tokyo 136-0075, Japan; 3 Department of Medical Genome Sciences, Graduate School of Frontier Science & Institute of Medical Science, University of Tokyo, 4-6-1 Shirokanedai, Minato-ku, Tokyo 108-8639, Japan; 4 Graduate Program of Biophysics and Biochemistry, Graduate School of Science, University of Tokyo, 4-6-1 Shirokanedai, Minato-ku, Tokyo 108-8639, Japan

## Abstract

**Background:**

The recent accumulation of closely related genomic sequences provides a valuable resource for the elucidation of the evolutionary histories of various organisms. However, although numerous alignment calculation and visualization tools have been developed to date, the analysis of complex genomic changes, such as large insertions, deletions, inversions, translocations and duplications, still presents certain difficulties.

**Results:**

We have developed a comparative genome analysis tool, named CGAT, which allows detailed comparisons of closely related bacteria-sized genomes mainly through visualizing middle-to-large-scale changes to infer underlying mechanisms. CGAT displays precomputed pairwise genome alignments on both dotplot and alignment viewers with scrolling and zooming functions, and allows users to move along the pre-identified orthologous alignments. Users can place several types of information on this alignment, such as the presence of tandem repeats or interspersed repetitive sequences and changes in G+C contents or codon usage bias, thereby facilitating the interpretation of the observed genomic changes. In addition to displaying precomputed alignments, the viewer can dynamically calculate the alignments between specified regions; this feature is especially useful for examining the alignment boundaries, as these boundaries are often obscure and can vary between programs. Besides the alignment browser functionalities, CGAT also contains an alignment data construction module, which contains various procedures that are commonly used for pre- and post-processing for large-scale alignment calculation, such as the split-and-merge protocol for calculating long alignments, chaining adjacent alignments, and ortholog identification. Indeed, CGAT provides a general framework for the calculation of genome-scale alignments using various existing programs as alignment engines, which allows users to compare the outputs of different alignment programs. Earlier versions of this program have been used successfully in our research to infer the evolutionary history of apparently complex genome changes between closely related eubacteria and archaea.

**Conclusion:**

CGAT is a practical tool for analyzing complex genomic changes between closely related genomes using existing alignment programs and other sequence analysis tools combined with extensive manual inspection.

## Background

Recently, many closely related prokaryotic and eukaryotic genome sequences have been determined, and detailed comparisons of these sequences are providing useful information regarding genomic evolution. To date, many alignment programs [1-13] and visualization tools [14-20] have been developed for large-scale genome comparisons. Typically, these tools are designed to extract conserved regions for identifying coding or regulatory regions, and they often assumed a simple collinear one-to-one correspondence between the sequences being compared. However, during prokaryotic evolution (and possibly also during eukaryotic evolution), crucial events, such as the acquisition or loss of functions that are related to pathogenicity and antibiotic resistance, symbiosis, and adaptation to new environments, are frequently associated with large chromosomal changes, such as insertions, deletions, substitutions, recombinations, and duplications of chromosomal segments, rather than with single nucleotide substitutions [21-23].

Previously, we conducted detailed comparisons of closely related microbial genomes in order to understand the mechanisms that generate such complex chromosomal changes [24-29]. For these studies, we required a visualization tool that provides both global views that show the correspondence between entire genomes and local views that show individual sequence alignments. We noticed that a combination of dotplot display and schematic alignment display is quite effective to understand complex chromosomal changes. In addition, the existence of characteristic structures, such as short tandem repeats, interspersed repetitive sequences, as well as changes in G+C content or codon usage bias provide valuable information regarding the processes that yield the observed genomic changes. Although some alignment visualization tools including PipMaker [14], ACT [19], GATA [18] and GenomeComp [16] provide views that are suitable for representing large-scale chromosomal changes, they are not adequate for the detailed analysis of complex changes in terms of the above demands.

In this report, we present a Comparative Genome Analysis Tool (CGAT) for comparisons of closely related genomes [see Additional file 1]. CGAT adopts a client-server architecture to provide both easy operability and advanced functionality, which is suitable for a collaborative research team that includes biologists who are willing to explore the genome alignment and informaticians who have some computer skills. CGAT visualizes precomputed homologous segment pairs between two genomes on both dotplot and alignment viewers. Users can explore the alignments on these viewers using scrolling and zooming functions and can compare the locations of several feature segments, such as repetitive structures identified on each genome. The preliminary versions of CGAT have been used in our internal research projects and have proved to be powerful in the analysis of apparently complex genome polymorphisms [24-29].

## Implementation

CGAT employs a client-server architecture, which consists of AlignmentViewer (client; a Java application) and DataServer (a set of Perl scripts). DataServer is a collection of data construction scripts and CGI scripts. AlignmentViewer visualizes the alignment data obtained from the server through the HTTP protocol or from the local file system when the server and client are installed on the same machine.

CGAT handles two types of data: sequence alignments between two genomes and feature segments identified on each genome. Feature segments are represented as the beginning and ending positions of the segments on each genome, and sequence alignments are represented as sets of two homologous segments. Basically, any program can be used to collect these data. CGAT DataServer contains a set of data construction scripts that offers a general framework for this task. In fact, the data construction process is almost completely automatic. In particular, when the genomic data to be compared are already stored in the MBGD database [30], CGAT automatically downloads data from the MBGD server before constructing the required data. Alternatively, users can prepare their own genomic data in the GenBank or FASTA format.

In the following sections, we first describe the data construction protocol implemented in DataServer and then introduce the AlignmentViewer program. In this work, we focus on prokaryotic genome comparisons, although in principle the program can also be applied to eukaryotic genome comparisons.

### Protocol for constructing genomic alignments

**Figure 1:**
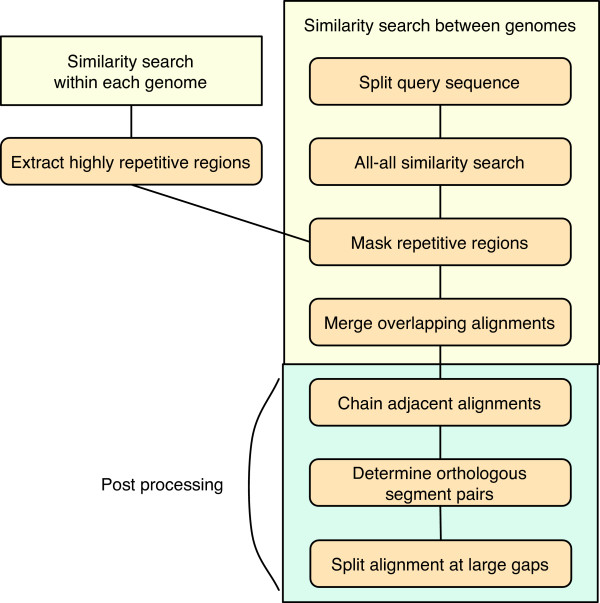
**Alignment construction protocol used in CGAT**. The actual alignment calculation is carried out at the "all-all similarity search" step using one of the alignment engines. For "similarity search within each genome" in the upper-left rectangle, the same protocol is used as in the frame labeled "similarity search between genomes". See the text for details.

The data construction module of CGAT DataServer defines its own protocol for calculating genomic alignments (Figure 1). However, it does not contain a program to calculate directly genome-to-genome alignments; instead, it uses various existing programs as alignment engines. By default, CGAT uses BLAST [31] or MegaBlast [32] to calculate alignments, but optionally it can incorporate FASTA [33], MUMmer [34], WABA [3], BLAT [5], BLASTZ [7], PatternHunter [6], CHAOS [8], GAME [12], and SSAHA [4]. In this study, we consider local alignment tools rather than global alignment tools, such as LAGAN [9] and AVID [11] because whole genome alignments generally contain rearrangements that are not handled well by global alignment tools.

For the analysis of long sequences, CGAT splits one of the genomic sequences into overlapping segments of appropriate length, performs an all-against-all comparison of the split sequences and the other genome, and then merges the resulting alignments that overlap with each other. The length of split sequences is determined for each program individually in consideration of the limitation of the program. Although this is a common protocol for calculating genome-scale alignments using traditional alignment programs, such as FASTA, it is still useful for aligning very long sequences using more modern programs.

**Figure 2:**
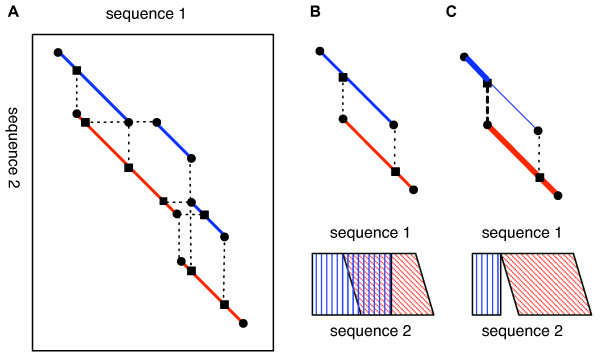
**Merging overlapping alignments**. (A) A directed acyclic graph (DAG) is constructed from the overlapping alignments, and the best path is then searched by a dynamic programming algorithm in order to resolve the overlap. A node of the DAG is every endpoint of block (filled circle) or internal point of block (filled square) that can be connected to some endpoint with a gap, and an edge of the DAG represents a block (solid line) or a gap (broken line). The blocks of two original alignments are indicated with red and blue lines, respectively. (B) A simplified example involving two overlapping alignment blocks. (C) The resolved alignment derived by taking the best path (thick lines).

A problem arises when merging overlapping alignments: the overlapping gapped alignments may not be consistent with each other, since they may be suboptimal or alternative optimal solutions. To solve this problem, CGAT decomposes a gapped alignment into a set of ungapped segment pairs (blocks) and compares the resulting sets of blocks. The sets of blocks should coincide with each other if and only if the overlapping gapped alignments are completely consistent. To resolve inconsistencies among alignments, CGAT constructs a directed acyclic graph (DAG) that consists of nodes that contain all the endpoints and some internal points of the blocks and edges representing blocks or gaps that connect two nodes in a gapped alignment (Figure 2), and finds a best-score path along the DAG using a dynamic programming algorithm.

In addition to solving the above "split and merge" alignment protocol, the overlap resolution procedure is, in some cases, also useful in simplifying the alignment output. For example, the output of PROmer, a program that is included in the MUMmer package and that performs translated sequence comparisons, often contains numerous overlapping alignments that correspond to the same alignment in different reading frames. In this type of case, the merging procedure resolves the overlap and simplifies the output.

Typically, the graph is sufficiently simple that the problem can be solved very quickly. However, sometimes the graph is very complex, especially when extremely highly repetitive sequences are present. To avoid this problem, the procedure extracts highly repetitive regions from each of the genomes by similarity searching prior to the main analysis (HighRep feature, see below), and eliminates the alignments that are covered in large part by these regions (Figure 1). This "repeat masking" is also important in simplifying the output because without this step, highly repetitive matches, the number of which is the square of the number of repetitive sequences in each sequence, would fill almost the entire region of the alignment and dotplot displays. Note that the repeat masking is carried out after the genome-to-genome comparison, and does not affect the alignments that are covered in small part by such repetitive regions.

### Post-processing of genome-to-genome alignments

In CGAT, each aligned segment pair is classified into one of four classes according to the best-hit relationships as follows: (1) orthologous segments; (2) segments duplicated only in the first genome; (3) segments duplicated only in the second genome; and (4) paralogous segments. An orthologous segment pair is operationally defined by a so-called 'bidirectional best hit', i. e. the segment pair having the best similarity score among the homologs of either of the segments. Classes 2 and 3 are defined by unidirectional best hits, i.e., the segment pair having the best score among the homologs of one of the segments. The other segment pairs are classified as paralogous segments.

The actual procedure for identifying the best-hit segment pairs is as follows: (1) all homologous segments are mapped onto each genome and the best similarity score is assigned to each region; and (2) an alignment that has a score >90% of the best score over at least 50% of the segment length is extracted as the best-scoring segment pair (note that the best score may be different among different regions). If the segment pair is the best-scoring pair for both of the genomes, then the segment pair is the bidirectional best pair.

Prior to the above classification process, CGAT attempts to create longer alignments by chaining non-overlapping adjacent alignments. This problem is similar to, but not identical to the overlapping resolution problem described above, since in this case only non-overlapping alignments are considered. We considered as being adjacent a pair of alignments in the same direction that are located within 50 kb in each of the sequences, and use the simple two-dimensional chain algorithm [35] (pp. 326–329) to find the optimal chain. The sum of the scores calculated by this procedure is assigned to each alignment and is used to identify orthologous segment pairs.

A similar alignment-chaining procedure is implemented in almost every program that performs large-scale alignments so as to make a longer alignment from initially shorter alignments. In contrast to these programs, CGAT does not try to create a longer alignment by concatenating the chained alignments. On the contrary, it splits the resulting alignments into smaller pieces in the final step when they contain large gaps (Figure 1), since eliminating large gaps from the alignments enhances presentation in AlignmentViewer. Nonetheless, AlignmentViewer can display these sequences as a contiguous long alignment by calculating alignment on the fly (see the section "CGAT AlignmentViewer" below).

### Collection of feature segments

Basically, the output of any DNA sequence analysis program that extracts sequence segments can be incorporated into CGAT as a feature segment; these analyses include pattern searching, weight matrix analysis, and detecting segments with atypical base composition.

Currently, we focus on the analysis of several types of repetitive structures that are frequently associated with the formation of genomic polymorphisms. The following programs are included in CGAT DataServer:

1) Interspersed highly repetitive regions (HighRep) analysis. CGAT uses a simple strategy to collect this type of repeat, in that it compares each genome to itself using the alignment protocol described above without the post-processing step (by default using MegaBlast as alignment engine), maps the resulting alignment onto each genome, and finally extracts the regions that are covered by alignments at least *T *times. The resulting regions can include various types of segments, such as tRNAs, insertion sequences (IS) or other mobile elements, and non-mobile repetitive elements, which include bacterial interspersed mosaic elements (BIMEs) [36], depending on the cutoff value *T*. CGAT collects regions using multiple *T *values and displays them with different colors in AlignmentViewer. The resulting set of HighRep segments is also used for masking repetitive regions in the alignment construction protocol described above.

2) Simple repeats (SimpleRep) analysis, which examines short tandem repeats with unit sizes of a few bases. It is well known that SimpleRep frequently yields polymorphisms for both eukaryotes and prokaryotes [37]. CGAT uses the Rep program (I. Uchiyama, unpublished) to collect this type of repeat. Rep uses a simple algorithm that is similar to XNU [38]; it searches high-scoring segment pairs (cutoff score *S*) between the same sequences shifted by *M *bp relative to each other, to identify repeats with unit of *M *bp, and outputs them if the number of repeats is at least *R*. By default, *M *is changed from 1 to 100 and *S *= 8 and *R *= 4 using the following scoring system: match +1, mismatch -3.

3) Direct or inverted repeats with an intervening sequence (DirRep/InvRep) analysis. This type of repeat is important, as it is frequently associated with insertion/deletion/inversion events. CGAT uses the Kmatch program (I. Uchiyama, unpublished) to collect this type of repeat. Kmatch uses the algorithm derived by Leung et al. [39] for hashing *k*-tuple words to search occurrences of almost identical sequences of at least *L *bp, while allowing *E *errors within an interval of up to *I*; the region is extended until the ratio of error becomes more than *R*. By default, we made the following settings: *L *= 30, *E *= 5, *R *= 0.15 and *I *= 5000 for DirRep and *L *= 24, *E *= 4, *R *= 0.15, and *I *= 5000 for InvRep.

4) Searching for known repetitive sequences. This approach, which is employed by the RepeatMasker program [40], is probably the most common way of identifying repetitive sequences in eukaryotic genomes. CGAT supports this type of analysis using an alignment engine (BLAST by default) when users carry a collection of repetitive sequences. For prokaryotic genomes, insertion sequences (IS) are the most common type of repetitive sequence, and the ISfinder database [41] represents a well-established collection of IS. Alternatively, one can use the GIB-IS database [42] as a downloadable IS database.

Genes are also considered to be special feature segments, and some attribute values can be assigned for each gene to be colored by AlignmentViewer. By default, CGAT uses the function categories assigned in the MBGD database [30] for coloring genes, although any program that characterizes gene or protein sequences can be used to assign attribute values. Currently, CGAT contains a program that calculates the codon usage bias defined by Karlin et al. [43] as well as a program that estimates G+C content at the third codon position (GC3); these values are useful for identifying candidates of horizontally transferred genes from distantly-related organisms.

### CGAT AlignmentViewer

**Figure 3:**
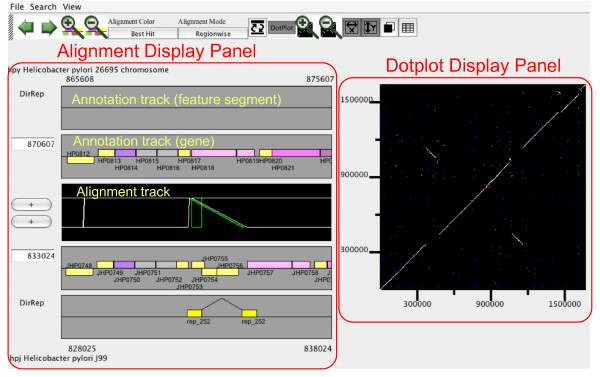
**CGAT AlignmentViewer**. The example shown is "insertion with long target duplication" [24], which was discovered during a comparison of two strains (26695 and J99) of *Helicobacter pylori*.

The data derived by the procedure described above are integrated and displayed in AlignmentViewer (Figure 3). The main window of AlignmentViewer consists of an alignment display panel (left) and a dotplot display panel (right), in addition to a common control panel (top). By default, the alignment and dotplot displays show precomputed alignments that are colored according to the four classes of best-hit relationships (see above). Optionally, the alignments can be colored according to the percentage identities. The alignment display panel contains three basic tracks: the central track (alignment track), which shows a graphical representation of the alignments, and the upper and lower tracks (annotation tracks), which show the annotation of the upper and lower genomes, respectively. More annotation tracks can be added to display the locations of several feature segments identified on each genome.

Users can change the current view on each display by pressing a scrolling or zooming button; these operations update both the alignment and dotplot displays in a coherent manner. Using the zooming function of the alignment display, users can change the scale from the entire genome level to the single nucleotide level. The scale of the dotplot display can also be changed independently of the alignment display. Furthermore, the scale of each axis can be changed independently; this feature is useful in visualizing the distribution of homologous regions of a specific segment on one genome against the entirety of the other genome (this point will be discussed further in the Results and discussion section).

Navigating the alignment space using the scrolling function is one of the key features of CGAT. In CGAT, the upper and lower sequences are considered as the reference and target sequences, respectively, and navigation is primarily a move along the reference sequence with a step size that depends on the current window size. Then the central position on the target sequence is automatically set according to the following rules: (1) if the next position is still in the current alignment, take the corresponding target position on that alignment; (2) if the next position is outside the current alignment but in some adjacent alignment, then set this alignment as the current one and take the corresponding target position on that alignment; (3) if there is no adjacent alignment, then search an orthologous alignment, and if there is an orthologous alignment, then set that alignment as the current one and take the corresponding target position on it; and (4) if there is no alignment, move the same extent as the reference sequence.

Basically, by continuous movement, users can navigate the entire genomes along the orthologous alignments. In addition, users can specify an arbitrary point on the dotplot display to move. In this manner, CGAT allows users to navigate easily within the entire alignment space.

**Figure 4:**
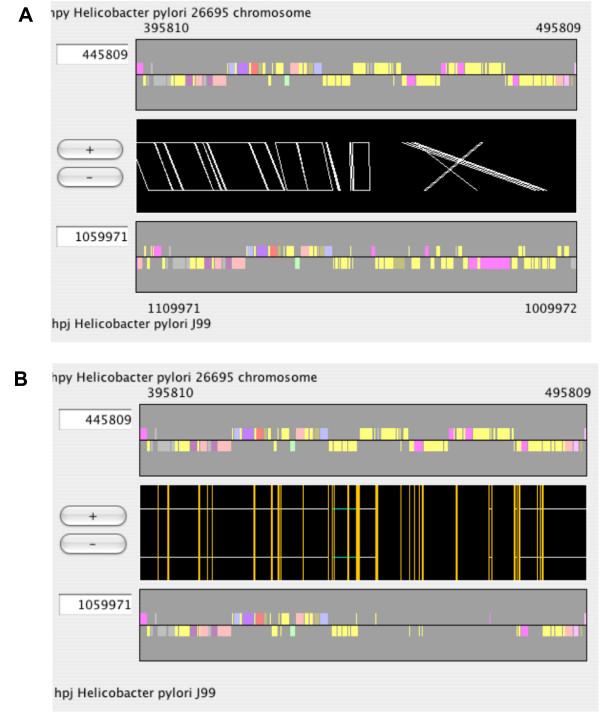
**Two modes of alignment display**. (A) The region-wise alignment mode, and (B) the reference-target alignment mode. These figures show the same region of the reference sequence (top). Note that in the reference-target alignment mode (B), the annotation track of the target sequence (bottom) is fragmented.

In CGAT, there are two modes in the alignment display (Figure 4). In the "region-wise alignment mode" (default, Figure 4A), alignments contained in the current region are displayed with a consecutive region specified in each genome. In the "reference-target alignment mode" (Figure 4B), every orthologous region of the target sequence is mapped as a fragment onto the reference sequence. The former mode is similar to ACT [19] and probably represents a more intuitive mode, whereas the latter mode is similar to PipMaker [14] and possibly represents a more informative mode for displaying large rearrangements; however it can show only orthologous matches.

In the region-wise mode, AlignmentViewer generally displays schematically the locations of the precomputed alignments within the region. However, when it displays an alignment at the nucleotide sequence level, AlignmentViewer dynamically realigns the displayed sequences using the dynamic programming algorithm for global alignment [44]. Therefore, in this mode, users can see the longer alignment beyond the boundary of the precomputed alignment. On the other hand, in the reference-target mode, AlignmentViewer uses the precomputed results to display the nucleotide sequence alignments.

Users can compare the locations of several feature segments, such as several types of repetitive segments, by loading them on the annotation tracks. In addition to retrieving the precomputed data from the server, AlignmentViewer can request the server to perform dynamical searches through the CGI interface. For example, users can search for sequences similar to their query sequence in each genome using BLAST or they can search for a motif using the regular expression pattern search. The results are displayed as feature segments on the annotation track in the alignment display panel. A list of locations for each feature segment can be shown in tabular format, which can be used to locate each segment on the alignment display.

## Results and discussion

The preliminary versions of CGAT [45] have already been used in our several research projects in microbial comparative genomics, including comparisons of *Helicobacter pylori *strains [24], *Pyrococcus horikoshii *and *P. abyssi *[25,26], *Neisseria meningitidis *strains, *N. meningitidis *strains and *N. gonorrhoeae *[27], and *Staphylococcus aureus *strains [28]. To highlight some unique functionalities of CGAT, we have chosen the example of a comparison of two strains of *H. pylori*. Further examples can be found on the project home page.

### *Comparison of *Helicobacter pylori *strains 26695 and J99*

*Helicobacter pylori *is the first bacterial species for which the genome sequences of two different strains were determined [46,47]. Comparative analysis of these sequences revealed several chromosomal rearrangements [47]. In further detailed analysis, Nobusato *et al*. found a characteristic pattern of polymorphisms in the *H. pylori *genomes, an insertion with long target duplication, which is frequently associated with the insertion of restriction-modification (RM) genes and which suggests a novel mechanism of gene mobility [24]. This pattern of polymorphisms is readily detected by CGAT with data from the direct repeat (DirRep) program loaded as feature segments (Figure 3). In this case, in addition to the DirRep track, the duplication can also be seen in the alignment track, in which green rectangles indicate that the aligned regions are duplicated only in the second (J99) genome. One can see the annotation of the inserted gene by moving the mouse cursor over it (Figure 3) and one can access the specified web server (by default the MBGD server) by clicking on it.

**Figure 5:**
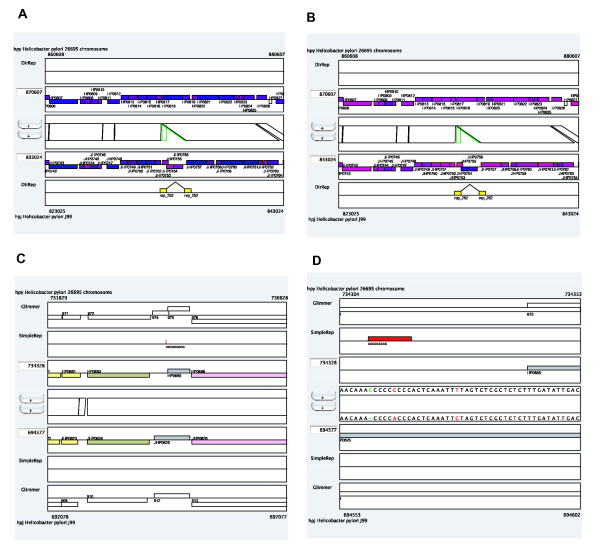
**AlignmentViewer display of the *Helicobacter pylori *data**. (A) The same example as that shown in Figure 3. Each gene is colored according to the codon usage bias with respect to the average of all genes, as introduced by Karlin et al. [43]. The highest bias is colored red, while the lowest bias is colored blue. (B) Similar to (A), except that each gene is colored according to the G+C content at the third codon position. The highest G+C content is colored red, while the lowest G+C content is colored blue. (C) ORF disruption caused by expansion of a simple repeat. The window size is 5000 bp. (D) Similar to (C), except using the window size of 50 bp.

The origin of the inserted genes is another interesting issue. Many of the strain-specific RM genes identified in the two *H. pylori *genomes were suggested to be horizontally transferred from distantly related organisms because of unusual codon usage bias and low G+C contents as well as the unusual topology of the phylogenetic trees [24]. In CGAT, codon usage bias and G+C content at the third codon position are pre-calculated as gene attribute values for each genome. Users can load one of these attributes to change gene colors (Figure 5A and 5B). In this case, the inserted gene showed high codon usage bias (Figure 5A) and low G+C content (Figure 5B), which suggest a horizontal transfer event.

Another interesting feature of the *H. pylori *genome is the abundance of simple repeat sequences [46,48], which are suggested to be involved in adaptive evolution by increasing genotypic variation due to slipped-strand mispairing [49]. The comparison of the genomes of the two strains revealed variations in the number of sequence repetitions [47]. Figure 5C and 5D shows the alignment display around the *fliP *genes (flagellar basal body protein; HP0685 and JHP0625) with simple repeat data (SimpleRep) and Glimmer prediction [50] loaded as feature segments. This clearly indicates that an increase in the length of a poly(C) tract results in a frame shift, which disrupts the reading frame of the *fliP *gene in strain 26695. It has been shown that this disruption results in loss of motility for this strain [51].

To facilitate the search for interesting structures associated with certain classes of genes or feature segments, CGAT provides several functions. By pressing the button farthest to the right on the control panel (or choosing 'View => Gene/Segment Data Table' from the menu), one can see the list of genes or specified feature segments in a tabular format. By clicking on each gene or segment on this table, one can change the current view to see alignments around the specified locus. In addition, users can filter genes or feature segments according to keyword or other parameter by choosing 'Search => Filter Gene/Segment' from the menu; in this function, only those segments that fulfill the specified conditions are displayed on the annotation track.

**Figure 6:**
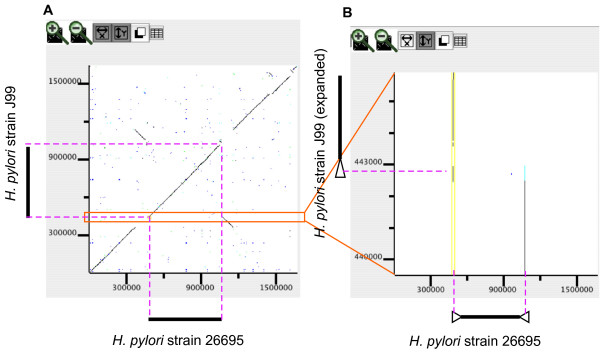
**Detecting repetitive structures by zooming only the y-axis in the dotplot display**. (A) Dotplot comparing the entire genomes of *H. pylori *strains 26695 (x-axis) and J99 (y-axis). (B) Dotplot with expanded y-axis scale clarifies the presence of inverted duplication at both ends of the rearranged segment in the 26695 genome (x-axis). Note that only the y-axis button is turned on in the control panel. The triangles drawn outside of the plot represent the duplicated structure.

While a pair of direct or inverted repeat sequences with a short spacer region can be easily visualized, as shown in Figure 3, it is more difficult to visualize repeat sequences that are farther apart. Indeed, a simple zoom-out operation to enlarge the displayed region shrinks everything and makes it difficult for users to grasp the relationship between distant points. In this type of case, independent scaling of the x-axis or y-axis in the dotplot display is useful. For example, the 26695 genome has a pair of inverted repeat sequences (named repeat 7 [46]) that are located at both ends of the rearranged segment that contains the putative replication terminus (thick lines in Figure 6), whereas that sequence is located at only one of the ends in the J99 genome, which partly accounts for the observed chromosomal inversion [47]. While this duplication is difficult to detect in the entire dotplot display (Figure 6A), it is clearly evident in the dotplot after zooming in on the y-axis (Figure 6B).

### Comparison of alignment engines

Another important feature of CGAT is to utilize several alignment programs as alignment engines, including BLASTN [31], MegaBlast[32], FASTA [33], MUMmer (NUCmer and PROmer) [34], WABA [3], BLAT [5], BLASTZ [7], PatternHunter (phn) [6], CHAOS [8], GAME [12], SSAHA, and SSAHA2 [4]. These programs use different algorithms or heuristics and different parameters and generally yield different results. Therefore, comparisons of alignments by multiple programs can be helpful in avoiding errors. In the following, we compare the performance characteristics of these alignment programs in terms of their usefulness as alignment engines in CGAT. For datasets, we used four pairs of closely related bacterial genomes: *Escherichia coli *K-12 [52] and O157:H7 [53], *Helicobacter pylori *26695 [46] and J99 [47], *Escherichia coli *K-12 [52] and *Salmonella enterica *serovar Typhi (*S. typhi*) CT18 [54], and *Bacillus subtilis *[55] and *Geobacillus kaustophilus *[56]. In this test, we ran each program with the default parameter set, with the aim of characterizing each program in a standard setting rather than fully investigating the potential performance through extensive changing of parameters. A similar, more extensive test was performed previously with a different set of programs using simulated data [57].

**Figure 7:**
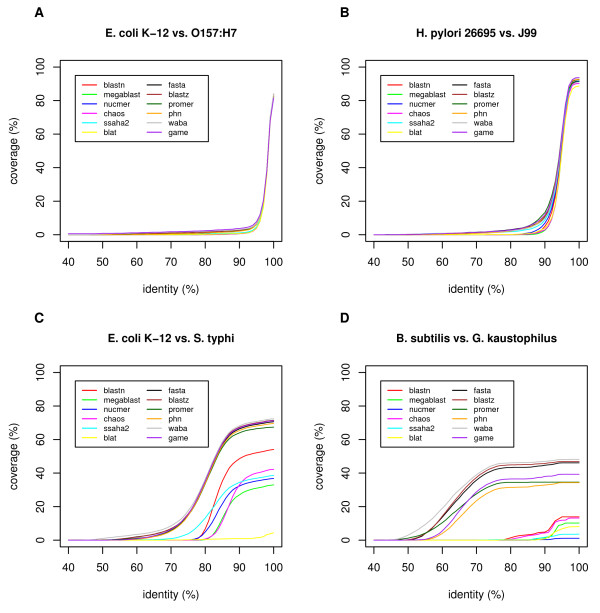
**Cumulative distribution of DNA sequence identities in orthologous alignments calculated by various programs**. Alignment coverage is defined as the sum of the lengths of orthologous alignments in both genomes divided by the sum of the entire chromosomal lengths of both genomes. In this analysis, each alignment longer than 500 bp is split into non-overlapping segments of 500 bp.

Figure 7 shows the cumulative distribution of the percentage identities of orthologous alignments in terms of alignment coverage, which is defined as the proportion of the lengths of the orthologous alignments to the entire chromosomal length. For example, in the comparison between the two *E. coli *strains (Figure 7A), most of the orthologous alignments had >98% identity and 80% or more of the entire chromosomes was covered by these alignments when using either of the programs. In the comparison between the two *H. pylori *strains (Figure 7B), the programs gave more variable results, although the identities of the alignments were still similarly distributed around 95% by all the programs. On the other hand, in the comparison between *E. coli *and *S. typhi*, a notable difference was observed (Figure 7C). WABA, BLASTZ, FASTA, GAME, PatternHunter (phn), and PROmer gave similar distributions centered around 80% identity and 60% or more coverage, whereas BLASTN, MegaBlast, CHAOS, SSAHA2, and NUCmer showed less sensitivity, and BLAT showed extremely low sensitivity. In the comparison of *B. subtilis *and *B. halodurans *(Figure 7D), in which the identities were centered around 65%, the difference between the programs was clearer. WABA, BLASTZ, and FASTA showed higher sensitivities than GAME, PROmer, and PatternHunter, and the other programs showed extremely low sensitivities. It seems that the more-sensitive programs, such as WABA, BLASTZ, and FASTA, are better (or required) for comparisons of weakly similar sequences.

**Figure 8:**
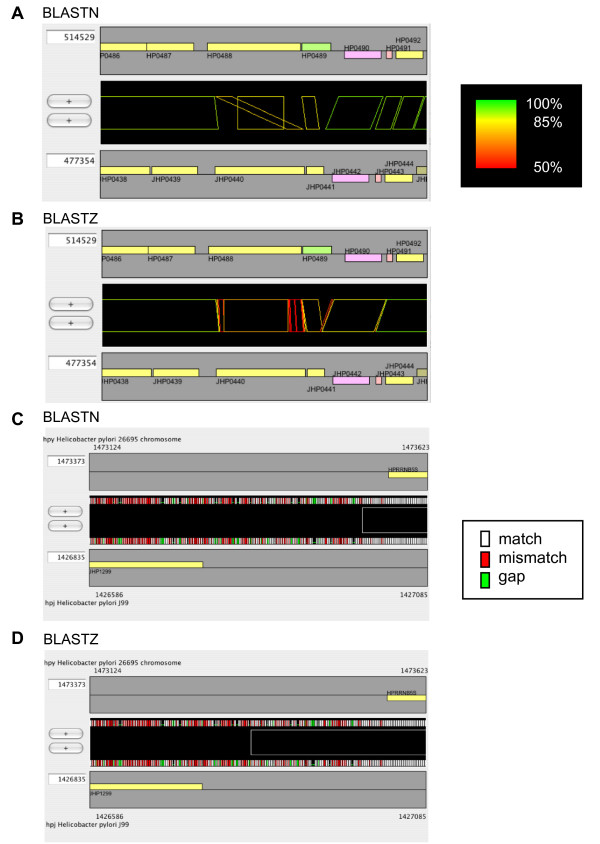
**Examples of differences between alignment engines**. Alignment of the same regions of *H. pylori *strains 26695 and J99, as calculated by BLASTN (A and C) and BLASTZ (B and D). In (A) and (B), each alignment is colored according to percentage identity, so that 100% is colored green, 85% is colored yellow, 50% is colored red, and the remaining identities are interpolated between these colors. In (C) and (D), the actual sequence alignment in this region is displayed with colors assigned to each character as follows: white, match; red, mismatch; green, gap.

However, comparative studies that yield precise information on elementary processes in evolution of genome structure are primarily those that compare genomes with nucleotide identities of ≥90%. In these types of studies, the sensitivity of the alignment program is not very important since the differences between the programs are small. Instead, the selectivity of the programs becomes important for identifying evolutionarily correct alignments. Unfortunately, there is usually a trade-off between sensitivity and selectivity, such that a more sensitive program may be less selective. Figure 8A and 8B shows an example, in which an apparent permutation found between HP0488 and its ortholog JHP0440 by BLASTN cannot be identified by BLASTZ, which was one of the most sensitive programs in the above tests. Similar problems are often encountered when using global alignment programs, and sensitive local alignment programs can also suffer from the same type of problem.

**Figure 9:**
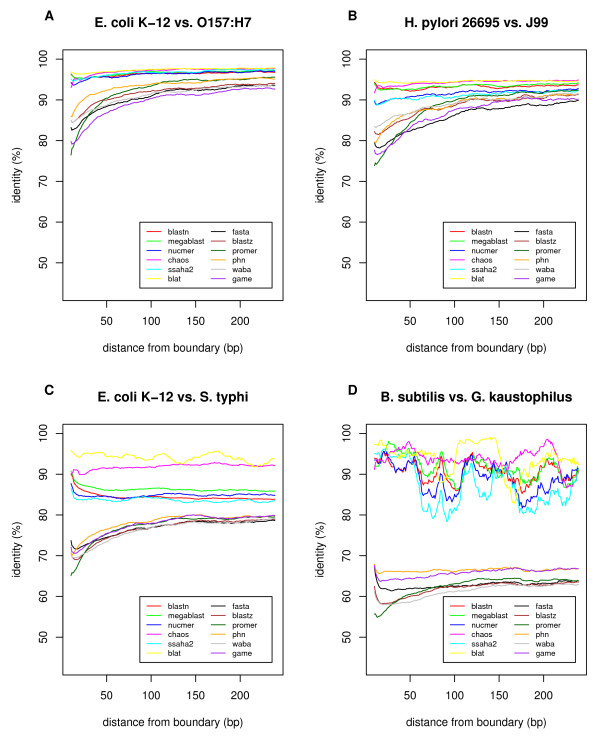
**Percentage identities at positions near the boundaries of the orthologous alignments calculated by various programs**. For each position at both ends of the orthologous alignments ≥ 500 bp, the number of matches and mismatches were counted and the average percentage identity was calculated using a window size of 21 bp (10 bp on either side) for smoothing. Gapped sites were eliminated from the alignments for the purpose of this calculation. The pairs of genomes to be compared are the same as those shown in Figure 7. The large variability observed among the "non-sensitive" set of programs in (D) is due to a lack of alignment data.

Alignment boundaries are often obscure and can vary among different programs. We examined the percentage identities at positions near the alignment boundaries, as calculated by each program (Figure 9), and found conspicuous differences between the sensitive methods (BLASTZ, FASTA, GAME, and PatternHunter) and the less-sensitive methods. In the former methods, identity substantially decreased near the alignment boundary, except in the comparison of the less-similar species of *Bacillus *and *Geobacillus*, whereas in the latter methods, this type of decay was not observed. This tendency, in addition to the increase in identity commonly observed at alignment boundaries, is probably due to the nature of the local alignment algorithm and is dependent upon the scoring systems and other parameters used, as well as the alignment algorithms or heuristics. In any case, it is difficult to say which program or set of parameters generally gives better alignment in terms of identifying true homology. In some cases, the aligned sequences share some conserved motifs that suggest that they are indeed homologous, while in other cases, these sequences may simply be non-homologous segments generated by a substitution or other rearrangement event. Therefore, it is recommended to careful researchers that they use multiple programs for choosing better alignments, thereby avoiding program-specific or parameter-specific errors.

In the region-wise mode of CGAT, the alignment between the displayed sequences is dynamically recalculated and displayed (Figure 8C and 8D), so that users can see the alignment beyond the boundaries of the precomputed ones. By simply reloading the alignments, one can compare alignments using different programs, as depicted in Figure 8. In addition, it may be helpful for users to load some feature segments calculated by other programs, such as a motif search program. In this way, CGAT allows users to validate carefully alignment quality.

## Conclusion

CGAT aims to help researchers to come to grips with the complex evolutionary changes that occur between closely related genomes through automated genome-to-genome alignments combined with extensive manual inspection. To achieve this goal, CGAT adopts a client-server architecture that comprises DataServer and AlignmentViewer, and has the following prominent features: (1) DataServer provides a general framework that defines a protocol for constructing large-scale genome alignments using various existing alignment programs; (2) DataServer also contains programs for collecting several feature segments, including several kinds of repetitive structures; (3) AlignmentViewer consists of an alignment display and a dotplot display with scrolling and zooming facilities, which are updated in a coherent fashion by user operations; (4) the alignment display can contain several annotation tracks that display precomputed or dynamically computed feature segments; (5) AlignmentViewer provides several functions that allow users to navigate efficiently through the alignment space and to filter information so as to focus on specific features; (6) in addition to displaying precomputed alignments, AlignmentViewer can calculate alignments between any specified regions on the fly, which enables users to validate or refine the precomputed alignments.

## Availability and requirements

**Project name: **CGAT

**Project home page: **

**Operating systems: **The client program is essentially platform-independent. The server program runs in the UNIX environment; it has been tested with Linux, Solaris, Darwin (Mac OSX), and Cygwin (for Windows).

**Programming languages: **Java (client) and Perl (server).

**License: **BSD.

This program is also available in its source code as additional file 1. For the latest version see the website.

## Abbreviations

CGAT: Comparative Genome Analysis Tool

DAG: directed acyclic graph

HighRep: highly repetitive region

SimpleRep: simple repeat

DirRep: direct repeat

InvRep: inverted repeat

IS: insertion sequence

RM: restriction-modification

## Authors' contributions

IU designed the software and developed the basic methods. IU and TH implemented the programs. IU conducted the analysis and wrote the manuscript. IK designed the practical projects using the software, which greatly influenced the program design. All of the authors read and approved the final manuscript.

## Supplementary Material

Additional File 1 Program source code. The source code of CGAT. The latest version can be found on the web site . Click here for file
