# Supplementary material for: CGAT: a comparative genome analysis tool for visualizing alignments in the analysis of complex evolutionary changes between closely related genomes
Source: BMC Bioinformatics. 2006 Oct 24;7:472. doi: 10.1186/1471-2105-7-472 (PMC1643837; doi:10.1186/1471-2105-7-472)
Supplement: Additional File 1 — Program source code. The source code of CGAT. The latest version can be found on the web site . [file 1471-2105-7-472-S1.tgz › CGAT/docs/book1.html]

CGAT User Manual

# CGAT User Manual

### Ikuo Uchiyama

National Institute for Basic Biology,
National Institutes of Natural Sciences

Copyright © 2006 by Ikuo Uchiyama

---

**Table of Contents**

What is CGAT ?

Installing CGAT
:   Requirements

    Download

    Install
    :   AlignmentViewer

        DataServer

The CGAT AlignmentViewer program
:   Starting AlignmentViewer
    :   Load data

    The main window of AlignmentViewer
    :   Control panel

        Alignment display panel

        Dotplot display panel

    Menu items
    :   File menu

        Search menu

        View menu

    Configuring AlignmentViewer

The CGAT DataServer package
:   Overview of CGAT DataServer
    :   Directory structure

    Database construction
    :   Overview of the data construction procedure

        Preparing genomic sequences

        Configuring database building procedure

        Start building

        Release data

    Web server set up

---

|  |  |  |
| --- | --- | --- |
|  |  | Next >>> |
|  |  | What is CGAT ? |
