# Supplementary material for: CGAT: a comparative genome analysis tool for visualizing alignments in the analysis of complex evolutionary changes between closely related genomes
Source: BMC Bioinformatics. 2006 Oct 24;7:472. doi: 10.1186/1471-2105-7-472 (PMC1643837; doi:10.1186/1471-2105-7-472)
Supplement: Additional File 1 — Program source code. The source code of CGAT. The latest version can be found on the web site . [file 1471-2105-7-472-S1.tgz › CGAT/docs/c15.html]

 What is CGAT ? 

| CGAT User Manual | | |
| --- | --- | --- |
| <<< Previous |  | Next >>> |

---

# What is CGAT ?

CGAT is a Comparative Genome Analysis Tool
that was developed for
detailed comparison of closely related bacterial-sized genomes, mainly for
analysis of complex evolutionary changes between two related
genomes. CGAT visualizes precomputed pairwise genome alignments
on both dotplot and alignment viewers. Users can put several
information on this alignment, such as existence of tandem repeats
or interspersed repetitive sequences and changes in codon usage bias,
to facilitate interpretation of the observed
genomic changes. Besides visualization functionalities,
CGAT also provides a general framework to process genome-scale
alignments using various existent alignment programs such as
BLAST/MegaBlast, FASTA, MUMmer, Blat and BlastZ.

Basically, CGAT employs a client-server architecture, which consists of
**AlignmentViewer** (client; a Java application) and
**DataServer**
(a set of Perl scripts).
The DataServer consists of data construction scripts and CGI scripts
and AlignmentViewer obtains the data from the server thorough the HTTP
protocol. AlignmentViewer can also obtain data from the local file system
when the server and client are installed on the same machine.

---

|  |  |  |
| --- | --- | --- |
| <<< Previous | Home | Next >>> |
| CGAT User Manual |  | Installing CGAT |
