# Supplementary material for: CGAT: a comparative genome analysis tool for visualizing alignments in the analysis of complex evolutionary changes between closely related genomes
Source: BMC Bioinformatics. 2006 Oct 24;7:472. doi: 10.1186/1471-2105-7-472 (PMC1643837; doi:10.1186/1471-2105-7-472)
Supplement: Additional File 1 — Program source code. The source code of CGAT. The latest version can be found on the web site . [file 1471-2105-7-472-S1.tgz › CGAT/docs/c22.html]

 Installing CGAT 

| CGAT User Manual | | |
| --- | --- | --- |
| <<< Previous |  | Next >>> |

---

# Installing CGAT

# Requirements

AlignmentViewer is written in Java and
should run on any machine supporting
Java (JDK 1.4 or higher).

DataServer is a set of Perl scripts
and assumes Unix environment with Perl
installed (version 5.8 or higher). It also assumes that the perl executable
is installed in the /usr/bin directory; please make
a symbolic link if it is located in a different directory.
The LWP (libwww-perl) module, which is downloadable from
the CPAN site,
is also required when you want to download data from the MBGD server.
DataServer also needs an HTTPD server for communicating with
the client program. In this manual, we assume that
Apache HTTPD version 2.0 or
more is installed on your server machine.

CGAT uses several programs for calculating alignments of two sequences
and identifying several feature segments on individual sequences.
Currently the following programs are supported:

**Table 1. Alignment programs currently supported by CGAT**

| program | URL |
| --- | --- |
| Blast/MegaBlast | http://www.ncbi.nlm.nih.gov/BLAST/ |
| Fasta | http://fasta.bioch.virginia.edu/ |
| MUMmer | http://mummer.sourceforge.net/ |
| Blat/WABA | http://www.cse.ucsc.edu/~kent |
| BlastZ | http://www.bx.psu.edu/miller\_lab/ |
| PatternHunter | http://www.bioinformaticssolutions.com/products/ph/ |
| CHAOS | http://lagan.stanford.edu/lagan\_web/index.shtml |
| GAME | http://cancer.informatics.indiana.edu/game/ |
| SSAHA | http://www.sanger.ac.uk/Software/analysis/SSAHA/ |

You should install one or more of the above programs on the server machine
before installing the CGAT DataServer. Please refer to the above URL for
installing individual programs. Basically, the DataServer assumes
at least the NCBI Blast/MegaBlast package is installed.

---

|  |  |  |
| --- | --- | --- |
| <<< Previous | Home | Next >>> |
| What is CGAT ? |  | Download |
