# Supplementary material for: CGAT: a comparative genome analysis tool for visualizing alignments in the analysis of complex evolutionary changes between closely related genomes
Source: BMC Bioinformatics. 2006 Oct 24;7:472. doi: 10.1186/1471-2105-7-472 (PMC1643837; doi:10.1186/1471-2105-7-472)
Supplement: Additional File 1 — Program source code. The source code of CGAT. The latest version can be found on the web site . [file 1471-2105-7-472-S1.tgz › CGAT/docs/x81.html]

 Download 

| CGAT User Manual | | |
| --- | --- | --- |
| <<< Previous | Installing CGAT | Next >>> |

---

# Download

The latest version of CGAT is available at
http://mbgd.genome.ad.jp/CGAT/.
You can download and install only the client program
(AlignmentViewer.jar)
or the full version including both the client and server programs
(CGAT.tar) depending on what you need.

- The client program (
   AlignmentViewer.jar).
  You can download and install only the client program
  if you intend to connect to the existing servers that you have
  already set up or to the default server
  (http://mbgd.genome.ad.jp/CGAT/) for trial.
- The client program as a Mac OSX application (
   AlignmentViewer.app).
  We recommend Mac OSX users to download
  this because it has been set up with increased memory
  allocation that solves the problem described in the next section.
- The full version of CGAT (
   CGAT.tgz),
  including both the client and server programs.
  You must install this package if you intend to analyze your own data locally.

---

|  |  |  |
| --- | --- | --- |
| <<< Previous | Home | Next >>> |
| Installing CGAT | Up | Install |
