# Supplementary material for: CGAT: a comparative genome analysis tool for visualizing alignments in the analysis of complex evolutionary changes between closely related genomes
Source: BMC Bioinformatics. 2006 Oct 24;7:472. doi: 10.1186/1471-2105-7-472 (PMC1643837; doi:10.1186/1471-2105-7-472)
Supplement: Additional File 1 — Program source code. The source code of CGAT. The latest version can be found on the web site . [file 1471-2105-7-472-S1.tgz › CGAT/docs/x101.html]

 Install 

| CGAT User Manual | | |
| --- | --- | --- |
| <<< Previous | Installing CGAT | Next >>> |

---

# Install

## AlignmentViewer

Basically, you need not particular operation after having downloaded
the program file (AlignmentViewer.jar) —
double click the icon (OSX or Windows) or type the java command
(Unix) to invoke the program. See the chapter
"
*The CGAT AlignmentViewer program*
"

However, in the default setting of the Java virtual machine (JVM),
you will be likely to encounter a memory allocation error
especially when aligning long sequences dynamically.
To avoid this problem, you need to increase the size of
the memory allocation pool for the JVM.

- Windows:
  Download the AlignmentViewer.jar file and
  do the following (see Figure 1).
  1. Create a shortcut of the AlignmentViewer.jar
     file (Right-click on the jar file ⇒
     **Create Shortcut**).
  2. Open the properties window of the shortcut (Right-click on the
     shortcut ⇒ **Properties**).
  3. Open the Shortcut tab and
     enter the java command in the **Target:** box as
     follows:
     [ **java -Xmx512m -jar "C:\Documents and Settings\..."** ],
     where **"C:\Documents and Settings\..."** is the
     original file location. This might be different in you situation,
     but do not modify the original name in anyway. Then, press "OK".
- Mac OSX:
  Download and use the OSX application version of AlignmentViewer
  (AlignmentViewer.app),
  instead of the AlignmentViewer.jar file.
- Linux/Unix:
  Download the AlignmentViewer.jar file and
  type **java -Xmx512m -jar AlignmentViewer.jar**
  to invoke the program.

**Figure 1. Increasing the size of the memory allocation pool of the JVM
for AlignmentViewer on Windows XP**

## DataServer

Copy the downloaded archive CGAT.tgz into
an appropriate directory and extract files by **tar** command,
which generates a directory named CGAT.
Enter this directory and invoke a configuration script
**SetUp.pl**.

```

tar cvfz CGAT.tgz
cd CGAT
SetUp.pl
```

SetUp.pl first asks you to set the CGAT\_HOME
environmental variable to the root of the generated directory tree
(hereafter we call this directory $CGAT\_HOME),
and subsequently it sets several command paths and compiles some programs.
SetUp.pl saves the CGAT\_HOME path in
scripts etc/cgat.sh for sh and bash
and etc/cgat.csh for csh and tcsh.
Before building the database or doing other operation, you must source the
above script.

```

# in csh or tcsh
source etc/cgat.csh

# in sh or bash
. etc/cgat.sh
```

We recommend you to add this command to your .cshrc script
(or .profile, .bashrc etc.)
(in this case, you must use the absolute path
rather than the relative path, of course).

The set up script tries to detect alignment programs available
on the server machine and installs detected programs
in the $CGAT\_HOME/build/align directory.
You might want to move some commands from the 
$CGAT\_HOME/build/align.in directory
or to remove some unnecessary commands manually.

Similarly, you might want to install manually some optional commands for
collecting feature segments from the $CGAT\_HOME/build/segment.opt
 directory. Note that in this case you must always set up
by yourself (if you wanted), since currently the set up script does not
try to check the availability of these commands automatically.
Currently, the following commands are available:

- **Glimmer**:
  Microbial gene-finding system available at
  http://www.cbcb.umd.edu/software/glimmer/.
  Currently, ver.2 is assumed by default, but you can use ver.3
  if you manually edit the commands/Glimmer.pl file.
  Before that, of course, you must correctly set up the glimmer program itself.
- **ISfind**:
  Searching an insertion sequence (IS) database by the BLAST program.
  The IS database should be a FASTA formatted sequence file with
  each comment line containing IS family name as
  ">*${ISname}*
  Fam:*${familyname}*",
  and the file should be installed as $CGAT\_HOME/database/isdb
  . See the IS Finder
  database for information about IS families. One can use the
  GIB-IS database
  available at DDBJ as a downloadable IS database. Download the table file
  (IS-table-text.txt) and run the following command:

  ```
  
$CGAT_HOME/build/bin/conv_GIB_IS.pl -nr IS-table-text.txt > $CGAT_HOME/database/isdb
  ```

---

|  |  |  |
| --- | --- | --- |
| <<< Previous | Home | Next >>> |
| Download | Up | The CGAT AlignmentViewer program |
