# Supplementary material for: CGAT: a comparative genome analysis tool for visualizing alignments in the analysis of complex evolutionary changes between closely related genomes
Source: BMC Bioinformatics. 2006 Oct 24;7:472. doi: 10.1186/1471-2105-7-472 (PMC1643837; doi:10.1186/1471-2105-7-472)
Supplement: Additional File 1 — Program source code. The source code of CGAT. The latest version can be found on the web site . [file 1471-2105-7-472-S1.tgz › CGAT/docs/c182.html]

 The CGAT AlignmentViewer program

| CGAT User Manual | | |
| --- | --- | --- |
| <<< Previous |  | Next >>> |

---

# The CGAT AlignmentViewer program

# Starting AlignmentViewer

On Windows or Mac OSX, double clicking on the "AlignmentViewer"
icon invokes the program.
On Unix systems, type the following command on the command line:

```

	java -jar AlignmentViewer.jar
```

However, when invoking with the above command, you will be
likely to encounter a memory allocation error especially when you
want to see an alignment of long sequences generated by dynamical
calculation.
To avoid this, we recommend you to increase the size of the memory
allocation pool for the Java virtual machine with
the -Xmx option.

```

	java -Xmx512m -jar AlignmentViewer.jar
```

## Load data

After running the program, first you must load data by choosing
**File ⇒ Load** from the menu.

**Figure 1. The menu window for specifying data to be loaded**

Server URL
:   Choose the server from which data are downloaded. By default, only
    the default CGAT server (running on the MBGD server) is registered.
    You can add more servers to this list in the Locations section
    of the Properties panel (**File ⇒ Properties**),
    if you or your colleagues have been setup an original new server.
    See the chapter "
    *The CGAT DataServer package*
    " for server setup and
    the "
    *Configuring AlignmentViewer*
    "section for properties setup.

Alignment Data
:   Choose the alignment data to be downloaded specified by the combination of
    organism names and a program name.
    Note that choosing the server URL above reset the list of
    available alignment data.
    If you have setup the data on the local file system that the AlignmentViewer
    program can directly accessed, and have correctly set the
    CGAT\_HOME variable in the Properties panel,
    you can also choose the data available
    on the local machine. These data are listed without the "(Server)" indication.

Gene Attribute
:   Choose the gene attribute data for coloring genes.

Feature Segment Data
:   Choose the feature segment data to be shown on the additional annotation
    tracks (see the section
    "*Alignment display panel*
    "). By default, maximally three types of segment data can
    be loaded at once, but the maximum number can be increased in
    the Properties panel.

---

|  |  |  |
| --- | --- | --- |
| <<< Previous | Home | Next >>> |
| Install |  | The main window of AlignmentViewer |
