# Supplementary material for: CGAT: a comparative genome analysis tool for visualizing alignments in the analysis of complex evolutionary changes between closely related genomes
Source: BMC Bioinformatics. 2006 Oct 24;7:472. doi: 10.1186/1471-2105-7-472 (PMC1643837; doi:10.1186/1471-2105-7-472)
Supplement: Additional File 1 — Program source code. The source code of CGAT. The latest version can be found on the web site . [file 1471-2105-7-472-S1.tgz › CGAT/docs/x227.html]

 The main window of AlignmentViewer

| CGAT User Manual | | |
| --- | --- | --- |
| <<< Previous | The CGAT AlignmentViewer program | Next >>> |

---

# The main window of AlignmentViewer

The main window of AlignmentViewer consists of four parts: a menu bar,
a control panel, an alignment display panel, and a dotplot display panel.
Both the alignment and dotplot displays show precomputed alignments.
Users can change the current view on each display by pressing a scrolling
or zooming button on the control panel. These operations update both
the alignment and dotplot displays in a coherent manner.

In AlignmentViewer, one of the compared sequences is considered as
the reference sequence and the other is considered as the
target sequence.
In the alignment display panel, the reference and the target sequences
are displayed on the upper and lower tracks, respectively,
and in the dotplot display panel, the reference and the target
sequences correspond to the x- and y-axes, respectively.
These sequences can be exchanged by **View**
⇒ **Exchange Reference Genome**.

**Figure 2. Main window of AlignmentViewer**

## Control panel

The control panel contains several buttons for controlling the current
view of both the alignment and dotplot displays.

**Figure 3. Control panel**

Move left/right
:   Move the current position to the left or right along the current alignment.

Zoom in/out
:   Zoom-in or -out the current view.

Alignment color
:   Toggle the alignment color mode between the "Best Hit" mode and
    the "Identity" mode.

    - Best Hit: white, orthologs; cyan, duplicated on the sequence 1;
      green, duplicated on the sequence 2; and blue, paralogs (by default).
    - Identity: red, 50%; yellow, 85%; and green, 100% (by default).

Alignment mode
:   Toggle the alignment mode between the "Regionwise" alignment mode and
    the "Reference-target" alignment mode.
    See the section "
    *Alignment display panel* ".

Exchange ref-target
:   Exchange the reference sequence and the target sequence.

Display dotplot
:   Toggle the status of the dotplot display panel between displayed and hidden.

Zoom in/out dotplot
:   Zoom-in or -out the dotplot display.

Zoomed axis
:   Specify the axis of the dotplot to be zoomed individually.

Change background
:   Toggle the background color of the alignment and dotplot displays
    between the dark color (black by default) and the light color
    (white by default).

Show annotation table
:   Show an annotation table for information on gene or feature segment.

**Figure 4. Alignment color modes. The Best-Hit mode (A),
and the Identity mode (B)**

## Alignment display panel

The alignment display panel contains three basic tracks: the central
tracks (alignment track) shows a graphical representation of the
alignments, and the upper and lower tracks (gene annotation tracks) show
the gene annotation of the upper and lower genomes, respectively.
More annotation tracks can be added to display the location of
several feature segments.

In addition, the "location fields" and the "strand switches" are on the left
side of the tracks.
The location field indicates the central coordinate of the current view,
and strand switch indicates the strand of each genome currently selected.
Users can jump to the desired location by entering a coordinate value
or a gene identifier (e.g. HP0812) in the location field, and can toggle
the strand direction by pressing the strand switch.

Some notes: 1) You must always enter the location of the reference genome
if you want to see the orthologous alignment around the specified locus.
If you want to specify the location in the current target genome,
you should exchanges the reference and the target genomes before
entering the position; 2) If you invert the reference genome by the
strand switch button, the direction of the subsequent movement is also
inverted. This means that when you press the 'move left' button,
the cursor in the dotplot display will move right, since the coordinate
of the dotplot display is not changed by this operation.

**Figure 5. Alignment display panel**

### Alignment modes

In CGAT, there are two modes in the alignment display:
"regionwise" alignment mode and "reference-target" alignment mode.
In the regionwise alignment mode, all alignments included in the current view
are displayed. Each alignment is represented as a trapezoid formed by
connecting the corresponding endpoints of the aligned region.
On the other hand, in the reference-target alignment mode, every
orthologous regions of the target sequence is mappend as a fragment
onto the reference sequence.

**Figure 6. The two alignment modes: the regionwise mode (A) and
the reference-target mode (B).**

### Alignment track

The alignment track displays precomputed alignments schematically.
Using the zooming buttons in the control panel, users can change
the scale from the entire genome level to the single nucleotide
level. In the nucleotide sequence alignment, mismatched site is colored
red and gapped site is colored green (by default).

**Figure 7. Zooming the alignment display**

In the regionwise alignment mode, left click on one of the alignment
regions (tetragons) sets the center of the selected alignment adjusted to
the center of the display.

By right click on the alignment track, a pop-up menu appears including
the following items:

- **View Re-Alignment**:
  Re-calculate alignment between the sequences within the current
  displayed region. This item appears only when the current
  displayed region is smaller than the specified length
  (Size of Realignment).
  See the "*Configuring AlignmentViewer*" section.
- **View Alignment**:
  Display the precomputed alignment under the clicked position.
- **Ident = ## %**:
  No action. The item is displayed only for showing the percent
  identity of the precomputed alignment under the clicked position.
- **Color Legend**:
  Display the color legend for the current alignment display.

### Annotation track

The annotation track displays several types of feature segments
identified in each genome.

By left click on one of the feature segments in the annotation track,
a pop-up menu appears:

- SegmentName:
  Open the web browser to access the web site,
  if an appropriate URL is defined on the server.
  Otherwise, no action is taken.
- **View Sequence: ## - ##**:
  Show the nucleotide sequence of the feature segment.

By right click on the annotation track, another pop-up menu appears:

- **Clicked Pos: ##** :
  No action. The item is displayed only for showing the clicked position.
- **Redraw (Set Center=##)** :
  Move the clicked position to the center of the display window.
- **Search Alignment ## - ##** :
  Search the set of precomputed alignments for those covering
  the region around the clicked position.
- **Color Legend**:
  Display the color legend for the current annotation display.

## Dotplot display panel

In the dotplot display panel, all alignments are plotted onto
the 2-dimensional space with the horizontal (X) and the vertical (Y)
axes representing the reference and the target sequences, respectively.
In this plot, the current scope, i.e. the region displayed in the alignment
display, is also shown as a rectangular region.
Users can move the current scope by clicking on any points in the plot
to move.

**Figure 8. Dotplot display panel**

In the dotplot display, independent scaling of the x-axis and y-axis
is possible. This feature is useful in visualizing the distribution
of homologous regions of a specific segment on one genome against
the entirety of the other genome.
In the following example, dotplot with expanded y-axis scale
clarifies the presence of inverted duplication at both ends
of the rearranged segment on the reference genome (x-axis).

**Figure 9. Dotplot display comparing the entire genomes (A) and
dotplot display with expanded y-axis scale**

---

|  |  |  |
| --- | --- | --- |
| <<< Previous | Home | Next >>> |
| The CGAT AlignmentViewer program | Up | Menu items |
