# Supplementary material for: CGAT: a comparative genome analysis tool for visualizing alignments in the analysis of complex evolutionary changes between closely related genomes
Source: BMC Bioinformatics. 2006 Oct 24;7:472. doi: 10.1186/1471-2105-7-472 (PMC1643837; doi:10.1186/1471-2105-7-472)
Supplement: Additional File 1 — Program source code. The source code of CGAT. The latest version can be found on the web site . [file 1471-2105-7-472-S1.tgz › CGAT/docs/x396.html]

 Menu items 

| CGAT User Manual | | |
| --- | --- | --- |
| <<< Previous | The CGAT AlignmentViewer program | Next >>> |

---

# Menu items

## File menu

### Load

Load data from the specified server or the local file system.
See the "*Starting AlignmentViewer*
" section.

### Print

Print out the image of the current alignment display.

### Properties

Configure user property parameters.
See the "*Configuring AlignmentViewer* " section.

### Exit

Exit the program.
This item appears only in the standalone application.
For applet version, you must exit the web browser to exit the program.

## Search menu

### Mark genes

Mark a specified set of genes.

An input form is opened for users to enter a list of gene names to be
marked. Gene names can be entered individually or be read from a file.
By pressing the **Search** button, gene names are
registered. Locations of the registered genes are indicated
on each axis of the dotplot display, and a new table window is
opened containing the information of the these genes.

Additional calls of this function will add genes to the existing list.
To remove genes from the list, enter the gene names to be removed and
press **Clear**.
To clear the entire list, press **All Clear**.

**Figure 10. Marking genes. Input form for entering genes (A) and the dotplot
with genes marked with triangles on each axis (B)**

### Filter Alignments

Filtering out the alignments that do not satisfy the specified
conditions. Alignments are filtered from both the alignment and dotplot
displays. A filter can be applied to one of the following fields:

- **Identity**: percentage identity; numeric.
- **Score**: similarity score; numeric.
- **Best Hit Status**:
  one of the following:
  3, orthologs (bidirectional best hits, BBH);
  2, duplicated only in the sequence-2;
  1, duplicated only in the sequence-1;
  0, paralogs.

For specification of the numeric field conditions, see the next subsection
"*Filter Genes/Segments*
"

Once filtered, a '\*' mark is displayed on the left side of the alignment
track (see Figure 11C).
Additional calls will add filters to the existing one.
For example, users can specify conditions such as "Best Hit Status is BBH
and Identity ≥ 90%" by two successive calls of this function.
To reset the filters, press the **Reset Alignment Filters**
button.

**Figure 11. Filtering alignments. The filtering condition window (A), and
alignment displays of before (B) and after (C) filtering (showing only BBH).**

### Filter Genes/Segments

Filtering out the genes or feature segments that do not satisfy the
specified conditions. A filter can be applied to any field of any
kind of segment that is currently loaded.

Kinds of conditions to be applied are different depending on
the types of the fields (numeric and string).
The following conditions are defined:

Numeric field
:   Available conditions are:
    **Between** (a ≤ x ≤ b),
    **Except Between**(x < a or b < x),
    **Less Than** (x < a),
    **Less Equal** (x ≤ a),
    **Equal** (x = a),
    **Greater Equal** (x ≥ a), and
    **Greater Than** (x > a),
    where a and/or b are values to be specified.
    To specify **Between** or **Except Between**,
    two values should be separated by a comma (e.g. "80,90").

String field
:   Available conditions are **Regex** (regular expression)
    and **Equal** (exact match).

Once filtered, a '\*' mark is displayed on the left side of the annotation track.
Additional calls will add filters to the existing one.
To reset the filter on the specified segment,
choose the segment type and press the **Reset This Filter**  button.
To reset all filters, press the **Reset All Filters** button.

**Figure 12. Filter genes/alignments**

### Dynamic Search

Search each genome for specific segments or sets of segments
dynamically, by calling a CGI script running on the server.
Results will be displayed in the additional annotation tracks,
or in the outermost pair of tracks (overwriting the existing data)
if the maximum number of segment tracks have already been used.

Currently, the following programs are available:

- **Blast**: BLAST similarity search for a nucleotide
  [blastn(direct) / tblastx(translation)] or a protein [tblastn] query sequence.
- **PatSearch**: Regular expression pattern search. See
  the document of the
  Java regex library.
- **DirRep**: Direct repeat search.
- **SimpleRep**: Simple repeat (or short tandem repeat) search.

## View menu

### Gene/Segment Data Table

Show table of genes or specified feature segment on the specified genome.
In the table, each row contains species name, the beginning position,
the ending position, strand direction, assigned color, and the name of
the segment.
Additional fields are added, if any, depending on the segment type.
By clicking a row of this table, the current scope is reset such that
the specified gene or segment is centered.

**Figure 13. Gene table**

### Sequence Window

Show nucleotide sequence of each genome within the current region.

### Exchange Reference Genome

Exchange the reference and the target sequences. Both the alignment and
dotplot displays are changed.

---

|  |  |  |
| --- | --- | --- |
| <<< Previous | Home | Next >>> |
| The main window of AlignmentViewer | Up | Configuring AlignmentViewer |
