# Supplementary material for: CGAT: a comparative genome analysis tool for visualizing alignments in the analysis of complex evolutionary changes between closely related genomes
Source: BMC Bioinformatics. 2006 Oct 24;7:472. doi: 10.1186/1471-2105-7-472 (PMC1643837; doi:10.1186/1471-2105-7-472)
Supplement: Additional File 1 — Program source code. The source code of CGAT. The latest version can be found on the web site . [file 1471-2105-7-472-S1.tgz › CGAT/docs/x528.html]

 Configuring AlignmentViewer 

| CGAT User Manual | | |
| --- | --- | --- |
| <<< Previous | The CGAT AlignmentViewer program | Next >>> |

---

# Configuring AlignmentViewer

Many properties of AlignmentViewer can be configured through property
parameters. These parameters can be changed on the properties window
(**File ⇒ Properties**). Configured properties
are saved in the user home directory.

In the properties window, the properties are categorized into the following
four sections.

1. **Locations**: Locations of data to be loaded.
   See "*Load data* "
   for loading data.
   - **CGAT\_HOME**:
     The server home directory containing the database.
     You can load data from the local file system,
     only when the CGAT database is installed in a directory
     accessible from the AlignmentViewer.
   - **Server URL**:
     Addresses of the CGAT servers. Multiple servers can be specified.
     You can choose which server to use when you load the data.
   - **Proxy URL**:
     Specify if your organization uses a proxy server.
     Please ask your network administrator.
2. **Color**: Color preferences.
   You can set two different sets of colors for the dark and light
   background color modes.
   - **Background**:
     The background color of both the alignment track and
     the dotplot display.
   - **Viewing Frame**:
     The color of the frame indicating the current scope in the dotplot
     display.
   - **Match/Mismatch/Gap**:
     Colors for displaying nucleotide sequence alignments in the
     annotation track.
   - **Identity**:
     Colors for drawing alignment regions according to the percentage
     identities (the identity color mode). Colors of the three points
     (highest, middle, and lowest percentages) can be specified, and
     the others are interpolated between these colors.
   - **Gene Attribute**:
     Colors for drawing genes in the annotation tracks using gene
     attribute values. Colors of the highest and lowest values can
     be specified, and the remaining values are interpolated between them.
3. **Panel**: Hight of the tracks and the maximum number of tracks.
   - **Maximum # of Segment Tracks**:
     Maximum number of feature segments to be loaded. Possibly,
     the height of the tracks should also be modified (lowered)
     to display all the information within the screen when you
     want to increase the number of segment tracks.
   - **Height of Alignment/Gene/Segment Area**:
     Hight of the tracks in the alignment display can be set individually
     for the alignment track, the gene annotation tracks and the
     feature segment tracks.
4. **Alignment**: Alignment parameters.
   - **Match/Mismatch/Open Gap/Extension Gap**:
     The scoring system for dynamically calculating alignments
     in the AlignmentViewer program.
   - **Size of Realignment**:
     Maximum length of the displayed region (scope) within which the
     re-alignment of the sequences is allowed by right click on the
     alignment track.

**Figure 14. Properties window**

---

|  |  |  |
| --- | --- | --- |
| <<< Previous | Home | Next >>> |
| Menu items | Up | The CGAT DataServer package |
