# Supplementary material for: CGAT: a comparative genome analysis tool for visualizing alignments in the analysis of complex evolutionary changes between closely related genomes
Source: BMC Bioinformatics. 2006 Oct 24;7:472. doi: 10.1186/1471-2105-7-472 (PMC1643837; doi:10.1186/1471-2105-7-472)
Supplement: Additional File 1 — Program source code. The source code of CGAT. The latest version can be found on the web site . [file 1471-2105-7-472-S1.tgz › CGAT/docs/c595.html]

 The CGAT DataServer package

| CGAT User Manual | | |
| --- | --- | --- |
| <<< Previous |  | Next >>> |

---

# The CGAT DataServer package

# Overview of CGAT DataServer

The CGAT DataServer package is a collection of data construction scripts and
CGI scripts. The data construction scripts run several programs to generate
comparison data to be visualized by the AlignmentViewer program.
AlignmentViewer can obtain data through the Internet using the CGI
interface implemented in the server package. Alternatively,
AlignmentViewer can also obtain data from the $CGAT\_HOME
directory when the server and client are installed on the same machine.

Data generated by CGAT DataServer can be classified into
the following three classes:

Alignment
:   A collection of locally aligned segment pairs between
    two genomes, which are displayed on both alignment and dotplot display
    panel in AlignmentViewer.

Feature Segments
:   A collection of feature segments in each genome,
    which can be displayed on the feature segment tracks in AlignmentViewer.

Gene Attribute
:   A value associated with each gene, which can be used to
    assign color to that gene in AlignmentViewer.

## Directory structure

**Table 1. List of directories on the $CGAT\_HOME directory**

| Directory | Contents |
| --- | --- |
| database | The current release of the database, which is accessed by the CGAT CGI scripts |
| database.work | Space for constructing the new database release |
| work | Workspace for database construction where many intermediate files are generated. |
| perllib | Perl libraries. |
| etc | Configuration tables etc. |
| build | Scripts for constructing the database |
| commands | Commands for identifying several feature segments on each genome. |
| htdocs | HTML documents and CGI scripts |
| client | Java source code of the client program (AlignmentViewer) |

**Table 2. List of directories on the database directory**

| Directory | Contents |
| --- | --- |
| genomes | Genomic sequences |
| genes | Gene information, including position table (tab subdirectory), nucleotide sequences (nt subdirectory), and amino acid sequences (aa subdirectory) |
| alignment | Collection of genome alignments calculated by several programs |
| segments | Collection of feature segments calculated by several programs |
| geneattr | Collection of gene attribute values calculated by several programs |

---

|  |  |  |
| --- | --- | --- |
| <<< Previous | Home | Next >>> |
| Configuring AlignmentViewer |  | Database construction |
