# Supplementary material for: CGAT: a comparative genome analysis tool for visualizing alignments in the analysis of complex evolutionary changes between closely related genomes
Source: BMC Bioinformatics. 2006 Oct 24;7:472. doi: 10.1186/1471-2105-7-472 (PMC1643837; doi:10.1186/1471-2105-7-472)
Supplement: Additional File 1 — Program source code. The source code of CGAT. The latest version can be found on the web site . [file 1471-2105-7-472-S1.tgz › CGAT/docs/x678.html]

 Database construction 

| CGAT User Manual | | |
| --- | --- | --- |
| <<< Previous | The CGAT DataServer package | Next >>> |

---

# Database construction

## Overview of the data construction procedure

Essentially, the data construction process consists of the following steps:

1. Prepare your genomic sequence data. If your sequences are already
   in the MBGD
   database, you can skip this step, because
   the procedure will automatically try to download missing data from MBGD.
2. Edit $CGAT\_HOME/etc/speclist
   to specify a set of species to be analyzed and a set of
   programs to be executed for each species set.
3. Run **$CGAT\_HOME/build/BuildAll.pl**.
   This script executes all procedures for building the database according
   to the $CGAT\_HOME/etc/speclist file,
   and eventually constructs data on the  $CGAT\_HOME/database.work directory.
4. Run **$CGAT\_HOME/build/Release.pl** to
   release the data from the $CGAT\_HOME/database.work
    directory to the $CGAT\_HOME/database
    directory.

## Preparing genomic sequences

Before running programs, you must prepare genome sequences data.
There are three possible ways.

1. If your sequences are already in the
   MBGD database,
   the simplest way to prepare data is to remember the abbreviated names of those
   genomes in the MBGD database (e.g. 'eco' for
   *Escherichi coli* K12).
   and use these names in the configuration table described
   in the next section. The build procedure automatically
   try to download missing data from MBGD.
   Note that the sequence name specified here is used throughout the system
   (hereafter we call it SPNAME).
2. Alternatively, if you have genomic data in GenBank format, you can use the
   $CGAT\_HOME/build/getDataFromGenBank.pl
   script to convert it to the CGAT database.
   The syntax of the command is

   **getDataFromGenBank.pl** *GBK\_FILE* *SPNAME*

   For example:

   ```
   
getDataFromGenBank.pl NC00913.gbk eco
   ```

   Here, you can use as SPNAME any name that is composed
   of alphanumeric characters, provided that it is unique.
3. Otherwise, you must prepare data by yourself. The data you should prepare
   are as follows:
   - Choose an appropriate unique name (SPNAME) that is
     composed of alphanumeric characters.
   - $CGAT\_HOME/database/genomes/SPNAME:
     a genomics sequence in fasta format.
   - $CGAT\_HOME/database/genes/aa/SPNAME:
     translated sequences of genes in fasta format
     (optional; if you want to calculate attribute values associated with
     protein sequences).
   - $CGAT\_HOME/database/genes/nt/SPNAME:
     nucleotide sequences of genes in fasta format
     (optional; if you want to calculate attribute values associated with
     nucleotide sequences).
   - $CGAT\_HOME/database/genes/tab/SPNAME:
     a tab-delimited table of genes
     containing the following information:
     beginning position, ending position,
     direction (1/-1), color code (= function category code; optional),
     the name of the gene, and the name of the product (optional).
     The beginning position must be smaller than the ending position
     even for the gene on the reverse chain.
     The file must begin with a header line containing a tab-delimited list of
     field names as follows,

     ```
     
#from	to	dir	color	name	product
     190	255	1	1	B0001	thr operon leader peptide
     337	2799	1	1	B0002	bifunctional aspartokinase I/homeserine dehydrogenase I
     2801	3733	1	1	B0003	homoserine kinase
     3734	5020	1	1	B0004	threonine synthase
     5234	5530	1	100	B0005	hypothetical protein
     ```

     By default color code is defined base on the MBGD function category.
     Specify '100' if you do not need to assign any color.
     You can change the color code by modifying
     $CGAT\_HOME/etc/colorTab/colorTab.gene.

## Configuring database building procedure

Next, you must prepare $CGAT\_HOME/etc/speclist file,
which contains information required for the database building procedure.
The following information should be specified in this file:

- A set of genomic sequences to be compared. If more than two species are
  specified, all-against-all pairwise comparison will be performed.
  Note that currently CGAT can handle only pairwise comparisons.
- A set of programs to be executed for each sequence set.

The speclist file consists of macro definition and
dataset definition sections, where macro definition must precede the
dataset definition. Syntax of the definition of macro variable is
as follows:

```

SET varname = value
```

A dollar sign followed by variable name, e.g. $varname, causes a
variable substitution, as usual.

The dataset definition section is a tab-delimited table, containing the
following fields: SPNAME\_LIST,
PROGRAM\_LIST,FLAG\_UPDATE,
and FLAG\_PUBLIC.
SPNAME\_LIST is a comma-delimited list specifying
a set of species to be compared.
PROGRAM\_LIST is a space-delimited list specifying
script files to be executed for each species set. The file names
are relative to the $CGAT\_HOME/build directory
and the wildcard characters such as '\*' can be used. For example,
align/\* specifies all (executable) files under the
$CGAT\_HOME/build/align directory.
FLAG\_UPDATE and FLAG\_PUBLIC
are flags specifying the data should be updated and the data should be
open to the public (through the CGI script), respectively.
The value should be 1 (yes) or 0 (no). Default value is 1.

The following is an example of the speclist file.
The file directs the build script to compare genome sequences
"hpy" and "hpj" by executing all programs for identifying
feature segments in the $CGAT\_HOME/build/segment
directory, followed by all programs for calculating gene attribute values
in the $CGAT\_HOME/build/geneattr directory,
followed by all programs for calculating alignment between the two genomes
n the $CGAT\_HOME/build/align directory.

```

####################
# macro definition
####################
SET AlignAll = align/*
SET SegmentAll = segment/* geneattr/*
####################
# dataset definition
####################
hpy,hpj	$SegmentAll $AlignAll
```

## Start building

Run **BuildAll.pl** under the $CGAT\_HOME/build
directory to execute building procedures. This script creates
$CGAT\_HOME/work directory if not exists, and move
to this directory, and then executes programs that have been specified in
the $CGAT\_HOME/etc/speclist file in the previous
section.

```

BuildAll.pl
```

## Release data

Before accessing the created database, you must run
**Release.pl** under the $CGAT\_HOME/build
directory to release the data from the $CGAT\_HOME/database.work
 directory to the $CGAT\_HOME/database
 directory.

```

Release.pl
```

---

|  |  |  |
| --- | --- | --- |
| <<< Previous | Home | Next >>> |
| The CGAT DataServer package | Up | Web server set up |
