# Supplementary material for: CGAT: a comparative genome analysis tool for visualizing alignments in the analysis of complex evolutionary changes between closely related genomes
Source: BMC Bioinformatics. 2006 Oct 24;7:472. doi: 10.1186/1471-2105-7-472 (PMC1643837; doi:10.1186/1471-2105-7-472)
Supplement: Additional File 1 — Program source code. The source code of CGAT. The latest version can be found on the web site . [file 1471-2105-7-472-S1.tgz › CGAT/docs/x796.html]

 Web server set up 

| CGAT User Manual | | |
| --- | --- | --- |
| <<< Previous | The CGAT DataServer package |  |

---

# Web server set up

AlignmentViewer can directly access the resulting
data file locally when the environmental variable $CGAT\_HOME
is correctly set. Therefore you can skip this section if you intend to
use AlignmentViewer only on the machine on which the database was
constructed.
On the other hand, if you want to allow access to the database from
various machines through the Internet, you must set up a web
server on the server machine.

If there is already a HTTP server running on the server, the simplest way
to add the CGAT service to the existing server is to create a symbolic link
to $CGAT\_HOME/htdocs/CGAT on a web site directory.
For example, if you have a public\_html directory
on your home directory as your own homepage, you can add CGAT service
to this page by creating the above symbolic link on this directory:

```

ln -s $CGAT_HOME/htdocs/CGAT ~/public_html
```

In this case, the URL for accessing the CGAT service is
http://YOUR\_MACHINE\_ADDRESS/~YOUR\_USER\_NAME/CGAT/,
where YOUR\_MACHINE\_ADDRESS and YOUR\_USER\_NAME should be replaced with
appropriate names.
If you are a Mac OSX user, you must have a Sites
instead of public\_html directory on your home directory
as your own home page.

Next you must edit the httpd.conf file to modify
the server setting.
The location of the httpd.conf file is system
dependent; on Mac OSX, user specific configuration can be described
in a separate file, /etc/httpd/users/USERNAME.conf,
where USERNAME should be replaced with your username.
In any way, this step usually requires administrator
privilege. Please ask an administrator of the machine if you do not
have the privilege.

The following is a configuration required for the above example
where the server is located on the public\_html directory:

```

<Directory /PATH_TO_YOUR_HOME_DIRECTORY/public_html>
    Options ExecCGI FollowSymLinks
    AddHandler cgi-script .cgi
    SetEnv CGAT_HOME /PATH_TO_CGAT_HOME
    SetEnv PERLLIB /PATH_TO_CGAT_HOME/perllib/
</Directory>
```

where /PATH\_TO\_YOUR\_HOME\_DIRECTORY and /PATH\_TO\_CGAT\_HOME should
be replaced with the absolute paths of the respective directories.

Possibly, you may need to load some additional Apache modules.
For example, the above setting contains the SetEnv directive which
requires the mod\_env module. If this module is not loaded in the default
setting, you must add the following line to the
httpd.conf file.

```

LoadModule env_module         MODULE_PATH/mod_env.so
```

where MODULE\_PATH should be replaced with a system dependent module path
(please ask your administrator).

Please remember that you must restart the HTTP server to reflect
the change after you modify the httpd.conf file.
Typically, this can be done by the command **apachectl restart** .
Mac OSX users can turn on/off the HTTP server by turning on/off,
respectively, the "Personal Web Sharing" service in the Sharing pane
opened through **Apple menu ⇒ System preference ⇒
Sharing**.

---

|  |  |  |
| --- | --- | --- |
| <<< Previous | Home |  |
| Database construction | Up |  |
