# Supplementary material for: CGAT: a comparative genome analysis tool for visualizing alignments in the analysis of complex evolutionary changes between closely related genomes
Source: BMC Bioinformatics. 2006 Oct 24;7:472. doi: 10.1186/1471-2105-7-472 (PMC1643837; doi:10.1186/1471-2105-7-472)
Supplement: Additional File 1 — Program source code. The source code of CGAT. The latest version can be found on the web site . [file 1471-2105-7-472-S1.tgz › CGAT/docs/manual.html]

CGAT User Manual

# CGAT User Manual

### Ikuo Uchiyama

National Institute for Basic Biology,
National Institutes of Natural Sciences

Copyright © 2006 by Ikuo Uchiyama

---

**Table of Contents**

What is CGAT ?

Installing CGAT
:   Requirements

    Download

    Install
    :   AlignmentViewer

        DataServer

The CGAT AlignmentViewer program
:   Starting AlignmentViewer
    :   Load data

    The main window of AlignmentViewer
    :   Control panel

        Alignment display panel

        Dotplot display panel

    Menu items
    :   File menu

        Search menu

        View menu

    Configuring AlignmentViewer

The CGAT DataServer package
:   Overview of CGAT DataServer
    :   Directory structure

    Database construction
    :   Overview of the data construction procedure

        Preparing genomic sequences

        Configuring database building procedure

        Start building

        Release data

    Web server set up

---

# What is CGAT ?

CGAT is a Comparative Genome Analysis Tool
that was developed for
detailed comparison of closely related bacterial-sized genomes, mainly for
analysis of complex evolutionary changes between two related
genomes. CGAT visualizes precomputed pairwise genome alignments
on both dotplot and alignment viewers. Users can put several
information on this alignment, such as existence of tandem repeats
or interspersed repetitive sequences and changes in codon usage bias,
to facilitate interpretation of the observed
genomic changes. Besides visualization functionalities,
CGAT also provides a general framework to process genome-scale
alignments using various existent alignment programs such as
BLAST/MegaBlast, FASTA, MUMmer, Blat and BlastZ.

Basically, CGAT employs a client-server architecture, which consists of
**AlignmentViewer** (client; a Java application) and
**DataServer**
(a set of Perl scripts).
The DataServer consists of data construction scripts and CGI scripts
and AlignmentViewer obtains the data from the server thorough the HTTP
protocol. AlignmentViewer can also obtain data from the local file system
when the server and client are installed on the same machine.

---

# Installing CGAT

## Requirements

AlignmentViewer is written in Java and
should run on any machine supporting
Java (JDK 1.4 or higher).

DataServer is a set of Perl scripts
and assumes Unix environment with Perl
installed (version 5.8 or higher). It also assumes that the perl executable
is installed in the /usr/bin directory; please make
a symbolic link if it is located in a different directory.
The LWP (libwww-perl) module, which is downloadable from
the CPAN site,
is also required when you want to download data from the MBGD server.
DataServer also needs an HTTPD server for communicating with
the client program. In this manual, we assume that
Apache HTTPD version 2.0 or
more is installed on your server machine.

CGAT uses several programs for calculating alignments of two sequences
and identifying several feature segments on individual sequences.
Currently the following programs are supported:

**Table 1. Alignment programs currently supported by CGAT**

| program | URL |
| --- | --- |
| Blast/MegaBlast | http://www.ncbi.nlm.nih.gov/BLAST/ |
| Fasta | http://fasta.bioch.virginia.edu/ |
| MUMmer | http://mummer.sourceforge.net/ |
| Blat/WABA | http://www.cse.ucsc.edu/~kent |
| BlastZ | http://www.bx.psu.edu/miller\_lab/ |
| PatternHunter | http://www.bioinformaticssolutions.com/products/ph/ |
| CHAOS | http://lagan.stanford.edu/lagan\_web/index.shtml |
| GAME | http://cancer.informatics.indiana.edu/game/ |
| SSAHA | http://www.sanger.ac.uk/Software/analysis/SSAHA/ |

You should install one or more of the above programs on the server machine
before installing the CGAT DataServer. Please refer to the above URL for
installing individual programs. Basically, the DataServer assumes
at least the NCBI Blast/MegaBlast package is installed.

---

## Download

The latest version of CGAT is available at
http://mbgd.genome.ad.jp/CGAT/.
You can download and install only the client program
(AlignmentViewer.jar)
or the full version including both the client and server programs
(CGAT.tar) depending on what you need.

- The client program (
   AlignmentViewer.jar).
  You can download and install only the client program
  if you intend to connect to the existing servers that you have
  already set up or to the default server
  (http://mbgd.genome.ad.jp/CGAT/) for trial.
- The client program as a Mac OSX application (
   AlignmentViewer.app).
  We recommend Mac OSX users to download
  this because it has been set up with increased memory
  allocation that solves the problem described in the next section.
- The full version of CGAT (
   CGAT.tgz),
  including both the client and server programs.
  You must install this package if you intend to analyze your own data locally.

---

## Install

### AlignmentViewer

Basically, you need not particular operation after having downloaded
the program file (AlignmentViewer.jar) —
double click the icon (OSX or Windows) or type the java command
(Unix) to invoke the program. See the chapter
"
*The CGAT AlignmentViewer program*
"

However, in the default setting of the Java virtual machine (JVM),
you will be likely to encounter a memory allocation error
especially when aligning long sequences dynamically.
To avoid this problem, you need to increase the size of
the memory allocation pool for the JVM.

- Windows:
  Download the AlignmentViewer.jar file and
  do the following (see Figure 1).
  1. Create a shortcut of the AlignmentViewer.jar
     file (Right-click on the jar file ⇒
     **Create Shortcut**).
  2. Open the properties window of the shortcut (Right-click on the
     shortcut ⇒ **Properties**).
  3. Open the Shortcut tab and
     enter the java command in the **Target:** box as
     follows:
     [ **java -Xmx512m -jar "C:\Documents and Settings\..."** ],
     where **"C:\Documents and Settings\..."** is the
     original file location. This might be different in you situation,
     but do not modify the original name in anyway. Then, press "OK".
- Mac OSX:
  Download and use the OSX application version of AlignmentViewer
  (AlignmentViewer.app),
  instead of the AlignmentViewer.jar file.
- Linux/Unix:
  Download the AlignmentViewer.jar file and
  type **java -Xmx512m -jar AlignmentViewer.jar**
  to invoke the program.

**Figure 1. Increasing the size of the memory allocation pool of the JVM
for AlignmentViewer on Windows XP**

---

### DataServer

Copy the downloaded archive CGAT.tgz into
an appropriate directory and extract files by **tar** command,
which generates a directory named CGAT.
Enter this directory and invoke a configuration script
**SetUp.pl**.

```

tar cvfz CGAT.tgz
cd CGAT
SetUp.pl
```

SetUp.pl first asks you to set the CGAT\_HOME
environmental variable to the root of the generated directory tree
(hereafter we call this directory $CGAT\_HOME),
and subsequently it sets several command paths and compiles some programs.
SetUp.pl saves the CGAT\_HOME path in
scripts etc/cgat.sh for sh and bash
and etc/cgat.csh for csh and tcsh.
Before building the database or doing other operation, you must source the
above script.

```

# in csh or tcsh
source etc/cgat.csh

# in sh or bash
. etc/cgat.sh
```

We recommend you to add this command to your .cshrc script
(or .profile, .bashrc etc.)
(in this case, you must use the absolute path
rather than the relative path, of course).

The set up script tries to detect alignment programs available
on the server machine and installs detected programs
in the $CGAT\_HOME/build/align directory.
You might want to move some commands from the 
$CGAT\_HOME/build/align.in directory
or to remove some unnecessary commands manually.

Similarly, you might want to install manually some optional commands for
collecting feature segments from the $CGAT\_HOME/build/segment.opt
 directory. Note that in this case you must always set up
by yourself (if you wanted), since currently the set up script does not
try to check the availability of these commands automatically.
Currently, the following commands are available:

- **Glimmer**:
  Microbial gene-finding system available at
  http://www.cbcb.umd.edu/software/glimmer/.
  Currently, ver.2 is assumed by default, but you can use ver.3
  if you manually edit the commands/Glimmer.pl file.
  Before that, of course, you must correctly set up the glimmer program itself.
- **ISfind**:
  Searching an insertion sequence (IS) database by the BLAST program.
  The IS database should be a FASTA formatted sequence file with
  each comment line containing IS family name as
  ">*${ISname}*
  Fam:*${familyname}*",
  and the file should be installed as $CGAT\_HOME/database/isdb
  . See the IS Finder
  database for information about IS families. One can use the
  GIB-IS database
  available at DDBJ as a downloadable IS database. Download the table file
  (IS-table-text.txt) and run the following command:

  ```
  
$CGAT_HOME/build/bin/conv_GIB_IS.pl -nr IS-table-text.txt > $CGAT_HOME/database/isdb
  ```

---

# The CGAT AlignmentViewer program

## Starting AlignmentViewer

On Windows or Mac OSX, double clicking on the "AlignmentViewer"
icon invokes the program.
On Unix systems, type the following command on the command line:

```

	java -jar AlignmentViewer.jar
```

However, when invoking with the above command, you will be
likely to encounter a memory allocation error especially when you
want to see an alignment of long sequences generated by dynamical
calculation.
To avoid this, we recommend you to increase the size of the memory
allocation pool for the Java virtual machine with
the -Xmx option.

```

	java -Xmx512m -jar AlignmentViewer.jar
```

---

### Load data

After running the program, first you must load data by choosing
**File ⇒ Load** from the menu.

**Figure 1. The menu window for specifying data to be loaded**

Server URL
:   Choose the server from which data are downloaded. By default, only
    the default CGAT server (running on the MBGD server) is registered.
    You can add more servers to this list in the Locations section
    of the Properties panel (**File ⇒ Properties**),
    if you or your colleagues have been setup an original new server.
    See the chapter "
    *The CGAT DataServer package*
    " for server setup and
    the "
    *Configuring AlignmentViewer*
    "section for properties setup.

Alignment Data
:   Choose the alignment data to be downloaded specified by the combination of
    organism names and a program name.
    Note that choosing the server URL above reset the list of
    available alignment data.
    If you have setup the data on the local file system that the AlignmentViewer
    program can directly accessed, and have correctly set the
    CGAT\_HOME variable in the Properties panel,
    you can also choose the data available
    on the local machine. These data are listed without the "(Server)" indication.

Gene Attribute
:   Choose the gene attribute data for coloring genes.

Feature Segment Data
:   Choose the feature segment data to be shown on the additional annotation
    tracks (see the section
    "*Alignment display panel*
    "). By default, maximally three types of segment data can
    be loaded at once, but the maximum number can be increased in
    the Properties panel.

---

## The main window of AlignmentViewer

The main window of AlignmentViewer consists of four parts: a menu bar,
a control panel, an alignment display panel, and a dotplot display panel.
Both the alignment and dotplot displays show precomputed alignments.
Users can change the current view on each display by pressing a scrolling
or zooming button on the control panel. These operations update both
the alignment and dotplot displays in a coherent manner.

In AlignmentViewer, one of the compared sequences is considered as
the reference sequence and the other is considered as the
target sequence.
In the alignment display panel, the reference and the target sequences
are displayed on the upper and lower tracks, respectively,
and in the dotplot display panel, the reference and the target
sequences correspond to the x- and y-axes, respectively.
These sequences can be exchanged by **View**
⇒ **Exchange Reference Genome**.

**Figure 2. Main window of AlignmentViewer**

---

### Control panel

The control panel contains several buttons for controlling the current
view of both the alignment and dotplot displays.

**Figure 3. Control panel**

Move left/right
:   Move the current position to the left or right along the current alignment.

Zoom in/out
:   Zoom-in or -out the current view.

Alignment color
:   Toggle the alignment color mode between the "Best Hit" mode and
    the "Identity" mode.

    - Best Hit: white, orthologs; cyan, duplicated on the sequence 1;
      green, duplicated on the sequence 2; and blue, paralogs (by default).
    - Identity: red, 50%; yellow, 85%; and green, 100% (by default).

Alignment mode
:   Toggle the alignment mode between the "Regionwise" alignment mode and
    the "Reference-target" alignment mode.
    See the section "
    *Alignment display panel* ".

Exchange ref-target
:   Exchange the reference sequence and the target sequence.

Display dotplot
:   Toggle the status of the dotplot display panel between displayed and hidden.

Zoom in/out dotplot
:   Zoom-in or -out the dotplot display.

Zoomed axis
:   Specify the axis of the dotplot to be zoomed individually.

Change background
:   Toggle the background color of the alignment and dotplot displays
    between the dark color (black by default) and the light color
    (white by default).

Show annotation table
:   Show an annotation table for information on gene or feature segment.

**Figure 4. Alignment color modes. The Best-Hit mode (A),
and the Identity mode (B)**

---

### Alignment display panel

The alignment display panel contains three basic tracks: the central
tracks (alignment track) shows a graphical representation of the
alignments, and the upper and lower tracks (gene annotation tracks) show
the gene annotation of the upper and lower genomes, respectively.
More annotation tracks can be added to display the location of
several feature segments.

In addition, the "location fields" and the "strand switches" are on the left
side of the tracks.
The location field indicates the central coordinate of the current view,
and strand switch indicates the strand of each genome currently selected.
Users can jump to the desired location by entering a coordinate value
or a gene identifier (e.g. HP0812) in the location field, and can toggle
the strand direction by pressing the strand switch.

Some notes: 1) You must always enter the location of the reference genome
if you want to see the orthologous alignment around the specified locus.
If you want to specify the location in the current target genome,
you should exchanges the reference and the target genomes before
entering the position; 2) If you invert the reference genome by the
strand switch button, the direction of the subsequent movement is also
inverted. This means that when you press the 'move left' button,
the cursor in the dotplot display will move right, since the coordinate
of the dotplot display is not changed by this operation.

**Figure 5. Alignment display panel**

---

#### Alignment modes

In CGAT, there are two modes in the alignment display:
"regionwise" alignment mode and "reference-target" alignment mode.
In the regionwise alignment mode, all alignments included in the current view
are displayed. Each alignment is represented as a trapezoid formed by
connecting the corresponding endpoints of the aligned region.
On the other hand, in the reference-target alignment mode, every
orthologous regions of the target sequence is mappend as a fragment
onto the reference sequence.

**Figure 6. The two alignment modes: the regionwise mode (A) and
the reference-target mode (B).**

---

#### Alignment track

The alignment track displays precomputed alignments schematically.
Using the zooming buttons in the control panel, users can change
the scale from the entire genome level to the single nucleotide
level. In the nucleotide sequence alignment, mismatched site is colored
red and gapped site is colored green (by default).

**Figure 7. Zooming the alignment display**

In the regionwise alignment mode, left click on one of the alignment
regions (tetragons) sets the center of the selected alignment adjusted to
the center of the display.

By right click on the alignment track, a pop-up menu appears including
the following items:

- **View Re-Alignment**:
  Re-calculate alignment between the sequences within the current
  displayed region. This item appears only when the current
  displayed region is smaller than the specified length
  (Size of Realignment).
  See the "*Configuring AlignmentViewer*" section.
- **View Alignment**:
  Display the precomputed alignment under the clicked position.
- **Ident = ## %**:
  No action. The item is displayed only for showing the percent
  identity of the precomputed alignment under the clicked position.
- **Color Legend**:
  Display the color legend for the current alignment display.

---

#### Annotation track

The annotation track displays several types of feature segments
identified in each genome.

By left click on one of the feature segments in the annotation track,
a pop-up menu appears:

- SegmentName:
  Open the web browser to access the web site,
  if an appropriate URL is defined on the server.
  Otherwise, no action is taken.
- **View Sequence: ## - ##**:
  Show the nucleotide sequence of the feature segment.

By right click on the annotation track, another pop-up menu appears:

- **Clicked Pos: ##** :
  No action. The item is displayed only for showing the clicked position.
- **Redraw (Set Center=##)** :
  Move the clicked position to the center of the display window.
- **Search Alignment ## - ##** :
  Search the set of precomputed alignments for those covering
  the region around the clicked position.
- **Color Legend**:
  Display the color legend for the current annotation display.

---

### Dotplot display panel

In the dotplot display panel, all alignments are plotted onto
the 2-dimensional space with the horizontal (X) and the vertical (Y)
axes representing the reference and the target sequences, respectively.
In this plot, the current scope, i.e. the region displayed in the alignment
display, is also shown as a rectangular region.
Users can move the current scope by clicking on any points in the plot
to move.

**Figure 8. Dotplot display panel**

In the dotplot display, independent scaling of the x-axis and y-axis
is possible. This feature is useful in visualizing the distribution
of homologous regions of a specific segment on one genome against
the entirety of the other genome.
In the following example, dotplot with expanded y-axis scale
clarifies the presence of inverted duplication at both ends
of the rearranged segment on the reference genome (x-axis).

**Figure 9. Dotplot display comparing the entire genomes (A) and
dotplot display with expanded y-axis scale**

---

## Menu items

### File menu

#### Load

Load data from the specified server or the local file system.
See the "*Starting AlignmentViewer*
" section.

---

#### Print

Print out the image of the current alignment display.

---

#### Properties

Configure user property parameters.
See the "*Configuring AlignmentViewer* " section.

---

#### Exit

Exit the program.
This item appears only in the standalone application.
For applet version, you must exit the web browser to exit the program.

---

### Search menu

#### Mark genes

Mark a specified set of genes.

An input form is opened for users to enter a list of gene names to be
marked. Gene names can be entered individually or be read from a file.
By pressing the **Search** button, gene names are
registered. Locations of the registered genes are indicated
on each axis of the dotplot display, and a new table window is
opened containing the information of the these genes.

Additional calls of this function will add genes to the existing list.
To remove genes from the list, enter the gene names to be removed and
press **Clear**.
To clear the entire list, press **All Clear**.

**Figure 10. Marking genes. Input form for entering genes (A) and the dotplot
with genes marked with triangles on each axis (B)**

---

#### Filter Alignments

Filtering out the alignments that do not satisfy the specified
conditions. Alignments are filtered from both the alignment and dotplot
displays. A filter can be applied to one of the following fields:

- **Identity**: percentage identity; numeric.
- **Score**: similarity score; numeric.
- **Best Hit Status**:
  one of the following:
  3, orthologs (bidirectional best hits, BBH);
  2, duplicated only in the sequence-2;
  1, duplicated only in the sequence-1;
  0, paralogs.

For specification of the numeric field conditions, see the next subsection
"*Filter Genes/Segments*
"

Once filtered, a '\*' mark is displayed on the left side of the alignment
track (see Figure 11C).
Additional calls will add filters to the existing one.
For example, users can specify conditions such as "Best Hit Status is BBH
and Identity ≥ 90%" by two successive calls of this function.
To reset the filters, press the **Reset Alignment Filters**
button.

**Figure 11. Filtering alignments. The filtering condition window (A), and
alignment displays of before (B) and after (C) filtering (showing only BBH).**

---

#### Filter Genes/Segments

Filtering out the genes or feature segments that do not satisfy the
specified conditions. A filter can be applied to any field of any
kind of segment that is currently loaded.

Kinds of conditions to be applied are different depending on
the types of the fields (numeric and string).
The following conditions are defined:

Numeric field
:   Available conditions are:
    **Between** (a ≤ x ≤ b),
    **Except Between**(x < a or b < x),
    **Less Than** (x < a),
    **Less Equal** (x ≤ a),
    **Equal** (x = a),
    **Greater Equal** (x ≥ a), and
    **Greater Than** (x > a),
    where a and/or b are values to be specified.
    To specify **Between** or **Except Between**,
    two values should be separated by a comma (e.g. "80,90").

String field
:   Available conditions are **Regex** (regular expression)
    and **Equal** (exact match).

Once filtered, a '\*' mark is displayed on the left side of the annotation track.
Additional calls will add filters to the existing one.
To reset the filter on the specified segment,
choose the segment type and press the **Reset This Filter**  button.
To reset all filters, press the **Reset All Filters** button.

**Figure 12. Filter genes/alignments**

---

#### Dynamic Search

Search each genome for specific segments or sets of segments
dynamically, by calling a CGI script running on the server.
Results will be displayed in the additional annotation tracks,
or in the outermost pair of tracks (overwriting the existing data)
if the maximum number of segment tracks have already been used.

Currently, the following programs are available:

- **Blast**: BLAST similarity search for a nucleotide
  [blastn(direct) / tblastx(translation)] or a protein [tblastn] query sequence.
- **PatSearch**: Regular expression pattern search. See
  the document of the
  Java regex library.
- **DirRep**: Direct repeat search.
- **SimpleRep**: Simple repeat (or short tandem repeat) search.

---

### View menu

#### Gene/Segment Data Table

Show table of genes or specified feature segment on the specified genome.
In the table, each row contains species name, the beginning position,
the ending position, strand direction, assigned color, and the name of
the segment.
Additional fields are added, if any, depending on the segment type.
By clicking a row of this table, the current scope is reset such that
the specified gene or segment is centered.

**Figure 13. Gene table**

---

#### Sequence Window

Show nucleotide sequence of each genome within the current region.

---

#### Exchange Reference Genome

Exchange the reference and the target sequences. Both the alignment and
dotplot displays are changed.

---

## Configuring AlignmentViewer

Many properties of AlignmentViewer can be configured through property
parameters. These parameters can be changed on the properties window
(**File ⇒ Properties**). Configured properties
are saved in the user home directory.

In the properties window, the properties are categorized into the following
four sections.

1. **Locations**: Locations of data to be loaded.
   See "*Load data* "
   for loading data.
   - **CGAT\_HOME**:
     The server home directory containing the database.
     You can load data from the local file system,
     only when the CGAT database is installed in a directory
     accessible from the AlignmentViewer.
   - **Server URL**:
     Addresses of the CGAT servers. Multiple servers can be specified.
     You can choose which server to use when you load the data.
   - **Proxy URL**:
     Specify if your organization uses a proxy server.
     Please ask your network administrator.
2. **Color**: Color preferences.
   You can set two different sets of colors for the dark and light
   background color modes.
   - **Background**:
     The background color of both the alignment track and
     the dotplot display.
   - **Viewing Frame**:
     The color of the frame indicating the current scope in the dotplot
     display.
   - **Match/Mismatch/Gap**:
     Colors for displaying nucleotide sequence alignments in the
     annotation track.
   - **Identity**:
     Colors for drawing alignment regions according to the percentage
     identities (the identity color mode). Colors of the three points
     (highest, middle, and lowest percentages) can be specified, and
     the others are interpolated between these colors.
   - **Gene Attribute**:
     Colors for drawing genes in the annotation tracks using gene
     attribute values. Colors of the highest and lowest values can
     be specified, and the remaining values are interpolated between them.
3. **Panel**: Hight of the tracks and the maximum number of tracks.
   - **Maximum # of Segment Tracks**:
     Maximum number of feature segments to be loaded. Possibly,
     the height of the tracks should also be modified (lowered)
     to display all the information within the screen when you
     want to increase the number of segment tracks.
   - **Height of Alignment/Gene/Segment Area**:
     Hight of the tracks in the alignment display can be set individually
     for the alignment track, the gene annotation tracks and the
     feature segment tracks.
4. **Alignment**: Alignment parameters.
   - **Match/Mismatch/Open Gap/Extension Gap**:
     The scoring system for dynamically calculating alignments
     in the AlignmentViewer program.
   - **Size of Realignment**:
     Maximum length of the displayed region (scope) within which the
     re-alignment of the sequences is allowed by right click on the
     alignment track.

**Figure 14. Properties window**

---

# The CGAT DataServer package

## Overview of CGAT DataServer

The CGAT DataServer package is a collection of data construction scripts and
CGI scripts. The data construction scripts run several programs to generate
comparison data to be visualized by the AlignmentViewer program.
AlignmentViewer can obtain data through the Internet using the CGI
interface implemented in the server package. Alternatively,
AlignmentViewer can also obtain data from the $CGAT\_HOME
directory when the server and client are installed on the same machine.

Data generated by CGAT DataServer can be classified into
the following three classes:

Alignment
:   A collection of locally aligned segment pairs between
    two genomes, which are displayed on both alignment and dotplot display
    panel in AlignmentViewer.

Feature Segments
:   A collection of feature segments in each genome,
    which can be displayed on the feature segment tracks in AlignmentViewer.

Gene Attribute
:   A value associated with each gene, which can be used to
    assign color to that gene in AlignmentViewer.

---

### Directory structure

**Table 1. List of directories on the $CGAT\_HOME directory**

| Directory | Contents |
| --- | --- |
| database | The current release of the database, which is accessed by the CGAT CGI scripts |
| database.work | Space for constructing the new database release |
| work | Workspace for database construction where many intermediate files are generated. |
| perllib | Perl libraries. |
| etc | Configuration tables etc. |
| build | Scripts for constructing the database |
| commands | Commands for identifying several feature segments on each genome. |
| htdocs | HTML documents and CGI scripts |
| client | Java source code of the client program (AlignmentViewer) |

**Table 2. List of directories on the database directory**

| Directory | Contents |
| --- | --- |
| genomes | Genomic sequences |
| genes | Gene information, including position table (tab subdirectory), nucleotide sequences (nt subdirectory), and amino acid sequences (aa subdirectory) |
| alignment | Collection of genome alignments calculated by several programs |
| segments | Collection of feature segments calculated by several programs |
| geneattr | Collection of gene attribute values calculated by several programs |

---

## Database construction

### Overview of the data construction procedure

Essentially, the data construction process consists of the following steps:

1. Prepare your genomic sequence data. If your sequences are already
   in the MBGD
   database, you can skip this step, because
   the procedure will automatically try to download missing data from MBGD.
2. Edit $CGAT\_HOME/etc/speclist
   to specify a set of species to be analyzed and a set of
   programs to be executed for each species set.
3. Run **$CGAT\_HOME/build/BuildAll.pl**.
   This script executes all procedures for building the database according
   to the $CGAT\_HOME/etc/speclist file,
   and eventually constructs data on the  $CGAT\_HOME/database.work directory.
4. Run **$CGAT\_HOME/build/Release.pl** to
   release the data from the $CGAT\_HOME/database.work
    directory to the $CGAT\_HOME/database
    directory.

---

### Preparing genomic sequences

Before running programs, you must prepare genome sequences data.
There are three possible ways.

1. If your sequences are already in the
   MBGD database,
   the simplest way to prepare data is to remember the abbreviated names of those
   genomes in the MBGD database (e.g. 'eco' for
   *Escherichi coli* K12).
   and use these names in the configuration table described
   in the next section. The build procedure automatically
   try to download missing data from MBGD.
   Note that the sequence name specified here is used throughout the system
   (hereafter we call it SPNAME).
2. Alternatively, if you have genomic data in GenBank format, you can use the
   $CGAT\_HOME/build/getDataFromGenBank.pl
   script to convert it to the CGAT database.
   The syntax of the command is

   **getDataFromGenBank.pl** *GBK\_FILE* *SPNAME*

   For example:

   ```
   
getDataFromGenBank.pl NC00913.gbk eco
   ```

   Here, you can use as SPNAME any name that is composed
   of alphanumeric characters, provided that it is unique.
3. Otherwise, you must prepare data by yourself. The data you should prepare
   are as follows:
   - Choose an appropriate unique name (SPNAME) that is
     composed of alphanumeric characters.
   - $CGAT\_HOME/database/genomes/SPNAME:
     a genomics sequence in fasta format.
   - $CGAT\_HOME/database/genes/aa/SPNAME:
     translated sequences of genes in fasta format
     (optional; if you want to calculate attribute values associated with
     protein sequences).
   - $CGAT\_HOME/database/genes/nt/SPNAME:
     nucleotide sequences of genes in fasta format
     (optional; if you want to calculate attribute values associated with
     nucleotide sequences).
   - $CGAT\_HOME/database/genes/tab/SPNAME:
     a tab-delimited table of genes
     containing the following information:
     beginning position, ending position,
     direction (1/-1), color code (= function category code; optional),
     the name of the gene, and the name of the product (optional).
     The beginning position must be smaller than the ending position
     even for the gene on the reverse chain.
     The file must begin with a header line containing a tab-delimited list of
     field names as follows,

     ```
     
#from	to	dir	color	name	product
     190	255	1	1	B0001	thr operon leader peptide
     337	2799	1	1	B0002	bifunctional aspartokinase I/homeserine dehydrogenase I
     2801	3733	1	1	B0003	homoserine kinase
     3734	5020	1	1	B0004	threonine synthase
     5234	5530	1	100	B0005	hypothetical protein
     ```

     By default color code is defined base on the MBGD function category.
     Specify '100' if you do not need to assign any color.
     You can change the color code by modifying
     $CGAT\_HOME/etc/colorTab/colorTab.gene.

---

### Configuring database building procedure

Next, you must prepare $CGAT\_HOME/etc/speclist file,
which contains information required for the database building procedure.
The following information should be specified in this file:

- A set of genomic sequences to be compared. If more than two species are
  specified, all-against-all pairwise comparison will be performed.
  Note that currently CGAT can handle only pairwise comparisons.
- A set of programs to be executed for each sequence set.

The speclist file consists of macro definition and
dataset definition sections, where macro definition must precede the
dataset definition. Syntax of the definition of macro variable is
as follows:

```

SET varname = value
```

A dollar sign followed by variable name, e.g. $varname, causes a
variable substitution, as usual.

The dataset definition section is a tab-delimited table, containing the
following fields: SPNAME\_LIST,
PROGRAM\_LIST,FLAG\_UPDATE,
and FLAG\_PUBLIC.
SPNAME\_LIST is a comma-delimited list specifying
a set of species to be compared.
PROGRAM\_LIST is a space-delimited list specifying
script files to be executed for each species set. The file names
are relative to the $CGAT\_HOME/build directory
and the wildcard characters such as '\*' can be used. For example,
align/\* specifies all (executable) files under the
$CGAT\_HOME/build/align directory.
FLAG\_UPDATE and FLAG\_PUBLIC
are flags specifying the data should be updated and the data should be
open to the public (through the CGI script), respectively.
The value should be 1 (yes) or 0 (no). Default value is 1.

The following is an example of the speclist file.
The file directs the build script to compare genome sequences
"hpy" and "hpj" by executing all programs for identifying
feature segments in the $CGAT\_HOME/build/segment
directory, followed by all programs for calculating gene attribute values
in the $CGAT\_HOME/build/geneattr directory,
followed by all programs for calculating alignment between the two genomes
n the $CGAT\_HOME/build/align directory.

```

####################
# macro definition
####################
SET AlignAll = align/*
SET SegmentAll = segment/* geneattr/*
####################
# dataset definition
####################
hpy,hpj	$SegmentAll $AlignAll
```

---

### Start building

Run **BuildAll.pl** under the $CGAT\_HOME/build
directory to execute building procedures. This script creates
$CGAT\_HOME/work directory if not exists, and move
to this directory, and then executes programs that have been specified in
the $CGAT\_HOME/etc/speclist file in the previous
section.

```

BuildAll.pl
```

---

### Release data

Before accessing the created database, you must run
**Release.pl** under the $CGAT\_HOME/build
directory to release the data from the $CGAT\_HOME/database.work
 directory to the $CGAT\_HOME/database
 directory.

```

Release.pl
```

---

## Web server set up

AlignmentViewer can directly access the resulting
data file locally when the environmental variable $CGAT\_HOME
is correctly set. Therefore you can skip this section if you intend to
use AlignmentViewer only on the machine on which the database was
constructed.
On the other hand, if you want to allow access to the database from
various machines through the Internet, you must set up a web
server on the server machine.

If there is already a HTTP server running on the server, the simplest way
to add the CGAT service to the existing server is to create a symbolic link
to $CGAT\_HOME/htdocs/CGAT on a web site directory.
For example, if you have a public\_html directory
on your home directory as your own homepage, you can add CGAT service
to this page by creating the above symbolic link on this directory:

```

ln -s $CGAT_HOME/htdocs/CGAT ~/public_html
```

In this case, the URL for accessing the CGAT service is
http://YOUR\_MACHINE\_ADDRESS/~YOUR\_USER\_NAME/CGAT/,
where YOUR\_MACHINE\_ADDRESS and YOUR\_USER\_NAME should be replaced with
appropriate names.
If you are a Mac OSX user, you must have a Sites
instead of public\_html directory on your home directory
as your own home page.

Next you must edit the httpd.conf file to modify
the server setting.
The location of the httpd.conf file is system
dependent; on Mac OSX, user specific configuration can be described
in a separate file, /etc/httpd/users/USERNAME.conf,
where USERNAME should be replaced with your username.
In any way, this step usually requires administrator
privilege. Please ask an administrator of the machine if you do not
have the privilege.

The following is a configuration required for the above example
where the server is located on the public\_html directory:

```

<Directory /PATH_TO_YOUR_HOME_DIRECTORY/public_html>
    Options ExecCGI FollowSymLinks
    AddHandler cgi-script .cgi
    SetEnv CGAT_HOME /PATH_TO_CGAT_HOME
    SetEnv PERLLIB /PATH_TO_CGAT_HOME/perllib/
</Directory>
```

where /PATH\_TO\_YOUR\_HOME\_DIRECTORY and /PATH\_TO\_CGAT\_HOME should
be replaced with the absolute paths of the respective directories.

Possibly, you may need to load some additional Apache modules.
For example, the above setting contains the SetEnv directive which
requires the mod\_env module. If this module is not loaded in the default
setting, you must add the following line to the
httpd.conf file.

```

LoadModule env_module         MODULE_PATH/mod_env.so
```

where MODULE\_PATH should be replaced with a system dependent module path
(please ask your administrator).

Please remember that you must restart the HTTP server to reflect
the change after you modify the httpd.conf file.
Typically, this can be done by the command **apachectl restart** .
Mac OSX users can turn on/off the HTTP server by turning on/off,
respectively, the "Personal Web Sharing" service in the Sharing pane
opened through **Apple menu ⇒ System preference ⇒
Sharing**.
