# Supplementary material for: CGAT: a comparative genome analysis tool for visualizing alignments in the analysis of complex evolutionary changes between closely related genomes
Source: BMC Bioinformatics. 2006 Oct 24;7:472. doi: 10.1186/1471-2105-7-472 (PMC1643837; doi:10.1186/1471-2105-7-472)
Supplement: Additional File 1 — Program source code. The source code of CGAT. The latest version can be found on the web site . [file 1471-2105-7-472-S1.tgz › CGAT/htdocs/CGAT/SequenceViewer.html]

SequenceViewer

Java Plug-In ‚ð—˜—p‚µ‚Ü‚·  


</COMMENT>
