# Supplementary material for: CGAT: a comparative genome analysis tool for visualizing alignments in the analysis of complex evolutionary changes between closely related genomes
Source: BMC Bioinformatics. 2006 Oct 24;7:472. doi: 10.1186/1471-2105-7-472 (PMC1643837; doi:10.1186/1471-2105-7-472)
Supplement: Additional File 1 — Program source code. The source code of CGAT. The latest version can be found on the web site . [file 1471-2105-7-472-S1.tgz › CGAT/htdocs/CGAT/index.html]

CGAT: Comparative Genome Analysis Tool


  

## A Comparative Genome Analysis Tool

  
**CGAT** is a Comparative Genome Analysis Tool that was depeloped for
detailed comparison of closely related bacterial-sized genomes.
CGAT visualizes precomputed pairwise genome alignments
on both dotplot and alignment viewers. Users can put several
information on this alignment, such as existence of tandem repeats
or interspersed repetitive sequences and changes in codon usage bias,
to facilitate interpretation of the observed genomic changes.
Besides visualization functionalities,
CGAT also provides a general framework to process genome-scale
alignments using various existing alignment programs.
CGAT employs a client-server architecture, which consists of
**AlignmentViewer** (client; a Java application) and **DataServer**
(a set of Perl scripts). The DataServer package contains data construction
scripts and CGI scripts and the AlignmentViewer program visualizes the
alignment data obtained from the server thorough the HTTP protocol.

---

- Invoke CGAT AlignmentViewer now
  - Start the applet version of CGAT AlignmentViewer (a demo version)
  - Download the software
    - The local install version of CGAT AlignmentViewer
      /
      [ For Mac OSX ]- The full version of CGAT
        including the DataServer package and the source code of AlignmentViewer
    - Documatations
      - CGAT manual (a single HTML file)- CGAT manual (multiple HTML flles)- CGAT manual (PostScript)
      - Examples
        - Insertions and duplications associated
          with a genome-wide inversion observed between
          *Neisseria meningitidis* Z2491 and MC58
        - Links to the related sites:
          - MBGD: Microbial genome database for comparative analysis- DomClust: Hierachical clustering program for orthologous grouping

          ---

          Please send questions and comments to Ikuo Uchiyama
          (uchiyama@nibb.ac.jp)
